# Results from the Survey of Antibiotic Resistance (SOAR) 2018–21 in Italy and Spain: data based on CLSI, EUCAST (dose-specific) and pharmacokinetic/pharmacodynamic (PK/PD) breakpoints

**DOI:** 10.1093/jac/dkaf283

**Published:** 2025-11-24

**Authors:** Didem Torumkuney, Rafael Canton, Cristina Pitart, Maurizio Sanguinetti, Chiara Vismara, Claudio Farina, Vittorio Sambri, Stephen Hawser, Anand Manoharan

**Affiliations:** Infectious Disease Research Unit, GSK, London, UK; Servicio de Microbiología, Hospital Universitario Ramon y Cajal and Instituto Ramón y Cajal de Investigación Sanitaria (IRYCIS), Madrid, Spain; CIBER de Enfermedades Infecciosas (CIBERINFEC), Madrid, Spain; Department of Microbiology, Hospital Clinic, University of Barcelona, ISGLOBAL, Barcelona, Spain; Dipartimento di Scienze di Laboratorio e Infettivologiche, Fondazione Policlinico Universitario A. Gemelli IRCCS, Roma, Italy; Chemical-Clinical and Microbiological Analysis, Azienda Socio-Sanitaria Territoriale (ASST) Grande Ospedale Metropolitano (GOM) Niguarda, Milano, Italy; USC Microbiologia e Virologia, Azienda Socio-Sanitaria Territoriale (ASST) Papa Giovanni XXIII, Bergamo, Italy; Department of Experimental, Diagnostic and Specialty Medicine-DIMES, Alma Mater Studiorum-University of Bologna, Bologna, Italy; IHMA Europe Sàrl, Rte. De I’Ile-au-Bois 1A, Monthey 1870, Switzerland; Infectious Diseases Medical & Scientific Affairs, GSK, Mumbai, India

## Abstract

**Objectives:**

To determine the antibiotic susceptibility of community-acquired respiratory tract infection (CA-RTI) *Streptococcus pneumoniae* and *Haemophilus influenzae* isolates from Italy and Spain, 2018–21.

**Methods:**

MICs were determined by CLSI broth microdilution, and susceptibility data were interpreted using CLSI, EUCAST and pharmacokinetic/pharmacodynamic (PK/PD) breakpoints.

**Results:**

A total of 77 *S. pneumoniae* and 249 *H. influenzae* were collected from Italy and 176 *S. pneumoniae* and 275 *H. influenzae* from Spain. Approximately 65% of pneumococci were penicillin-susceptible by CLSI oral or EUCAST low-dose breakpoints; by EUCAST high-dose or CLSI intravenous administration, 92.2%/95.5% (Italy/Spain) were susceptible. In Italy, 71.4% to 89.6% susceptibility (CLSI) was observed to amoxicillin/clavulanic acid, amoxicillin and most cephalosporins. Cefaclor, tetracyclines, macrolides and trimethoprim/sulfamethoxazole were less active against Italian (51.9%–67.5% susceptible) versus Spanish (67.0%–81.2% susceptible) isolates. The most potent antibiotics were fluoroquinolones (≥99.4%, excluding EUCAST low-dose). Most *H. influenzae* isolates were β-lactamase negative with a few ampicillin-resistant isolates (CLSI: 0.5% Italy, 3.1% Spain). Rates of β-lactamase positivity were 14.1% (Italy) and 17.1% (Spain). Susceptibility was >90% (CLSI) except for ampicillin (82.7% Italy, 81.1% Spain) and trimethoprim/sulfamethoxazole (71.1% Italy, 68.0% Spain). Susceptibility by EUCAST was similar to CLSI, except for cefuroxime (82.3% Italy, 87.3% Spain susceptible, increased exposure versus 100% by CLSI).

**Conclusions:**

Susceptibility of *S. pneumoniae* to many antibiotics was low in both countries, with susceptibility > 90% observed only with high-dose penicillin and fluoroquinolones (CLSI and EUCAST) and high-dose amoxicillin or amoxicillin/clavulanic acid (PK/PD). Higher susceptibility was seen with *H. influenzae* in both countries. Continued surveillance of antimicrobial resistance is important for guiding therapy of CA-RTIs.

## Introduction

Community-acquired respiratory tract infections (CA-RTIs) are an important world health problem that, if treated incorrectly, or in patients with comorbidities, can result in hospitalization, with a third of patients with community-acquired pneumonia dying within 12 months after being discharged from hospital.^1^ Comorbidities, age and other risk factors might have also impacted the mortality rate.^1^ Treatment of CA-RTIs is reliant on empiric antibiotic therapy through the use of national and international guidelines.^2^ A recent study investigating trends in antibiotic prescribing in primary care and its association with drug-resistant microorganisms found Italy and Spain to have the highest prevalence of sentinel drug-resistant organisms, including *Streptococcus pneumoniae*, and the highest volume of antibiotic use in primary care.^3^ The extensive use of antibiotics is associated with resistance development.^4^

*S. pneumoniae* and *Haemophilus influenzae* are the major bacteria associated with CA-RTIs.^5,6^ Both pathogens have shown increasing resistance to first-line antibiotics such as penicillin and ampicillin.^7,8^ As rates of resistance vary over time and from country to country, up-to-date surveillance data are essential to guide local antibiotic policies,^9^ including appropriate antibiotic prescribing and guideline formulation.^10^

The Survey of Antibiotic Resistance (SOAR), an international antibiotic resistance surveillance study, focuses on key respiratory pathogens that cause community-acquired infections and has been running since 2002 in the Middle East, Africa, Latin America, Asia-Pacific, Europe and the Commonwealth of Independent States countries.^11^ For this study, data from hospitals in Italy and Spain have been analysed to provide a picture of the current state of antibiotic susceptibility of *S. pneumoniae* and *H. influenzae* associated with CA-RTIs in these two countries. This is the first time these two countries have been included in the SOAR surveillance programme.

## Materials and methods

### Ethics

SOAR studies are not human subject studies. During the study, only microorganisms were examined.

### Collaborating centres

Isolates were provided between 2018 and 2021 from four sites in Italy [Azienda Socio-Sanitaria Territoriale (ASST) Papa Giovanni XXIII, Bergamo; Laboratorio Unico del Centro Servizi, Pievesestina; Azienda Socio-Sanitaria Territoriale (ASST) Grande Ospedale Metropolitano (GOM) Niguarda, Milano; and Policlinico Agostino Gemelli, Roma] and three sites in Spain (Hospital Clinic, Barcelona; Hospital Universitario Ramon y Cajal, Madrid; and Hospital Universitario Virgen de la Macarena, Seville).

### Clinical isolates

Isolates of *H. influenzae* and *S. pneumoniae* from CA-RTIs (isolated within 48 h of hospitalization) were sent to a central laboratory (IHMA Europe, Monthey, Switzerland), where they were sub-cultured and re-identified. *H. influenzae* were re-identified by MALDI-TOF MS methodology, and *S. pneumoniae* identity was confirmed by optochin susceptibility and bile solubility. β-Lactamase production was determined for each *H. influenzae* isolate by a chromogenic cephalosporin (nitrocefin) disc method. Duplicate isolates from the same patient were not accepted.

### Susceptibility testing

Isolates were evaluated for antibiotic susceptibility using broth microdilution methodology as recommended by CLSI.^12^ Amoxicillin, amoxicillin/clavulanic acid (2:1 ratio as per CLSI guidelines^12,13^), amoxicillin/clavulanic acid (fixed clavulanic acid at 2 mg/L as per EUCAST guidelines^14^), azithromycin, cefaclor, cefdinir, cefixime, cefotaxime, cefpodoxime, ceftibuten, ceftriaxone, cefuroxime, clarithromycin, levofloxacin, moxifloxacin and trimethoprim/sulfamethoxazole (1:19 ratio) were tested against both respiratory pathogens. In addition, doxycycline, erythromycin and penicillin were tested against *S. pneumoniae* only, and ampicillin was tested against *H. influenzae* only. Susceptibility to the study drugs was calculated based on CLSI breakpoints and EUCAST (dose-specific) breakpoints.^12–14^ These breakpoints are shown in Tables 1 and 2. To fully assess antibiotics where high-dose therapies are available, susceptibility using EUCAST criteria was also calculated by combining percentage susceptible and percentage ‘susceptible, increased exposure’ into the susceptible category.^14^ The antibiotics with high-dose availability assessed in this way were as follows: amoxicillin (0.75–1 g oral, 3× daily), amoxicillin/clavulanic acid (0.875 g amoxicillin/0.125 g clavulanic acid oral, 3× daily), ampicillin (2 g IV, 4× daily), penicillin (2.4 g IV, 2 MU 4–6× daily), ceftriaxone (2 g IV, 2× daily), clarithromycin (0.5 g oral, 2× daily), erythromycin (1 g oral or IV, 4× daily), levofloxacin (0.75 g oral 2× daily, or 0.4 g IV 3× daily) and trimethoprim/sulfamethoxazole (0.24 g trimethoprim/1.2 g sulfamethoxazole oral or IV, 2× daily).^14^ A further analysis of amoxicillin susceptibility based on a higher dosage of 4 g amoxicillin per day and a higher dosage of 4 g amoxicillin/0.25 g clavulanic acid per day [using amoxicillin/clavulanic acid (2:1) MICs] using a published pharmacokinetic/pharmacodynamic (PK/PD) susceptible breakpoint of ≤4 mg/L (amoxicillin component) was also performed.^15^ PK/PD breakpoints were also used in the analysis (Table 3).

**Table 1.** CLSI MIC breakpoints (mg/L) used for *S. pneumoniae* and *H. influenzae* isolates

	*S. pneumoniae*			*H. influenzae*		
Antimicrobial	S	I	R	S	I	R
Amoxicillin	≤2	4	≥8	—	—	—
Amoxicillin/clavulanic acid (2:1)^a^	≤2	4	≥8	≤2	4	≥8
Ampicillin	NT	NT	NT	≤1	2	≥4
Azithromycin	≤0.5	1	≥2	≤4	—	—
Cefaclor	≤1	2	≥4	≤8	16	≥32
Cefdinir	≤0.5	1	≥2	≤1	—	—
Cefixime	—	—	—	≤1	—	—
Cefotaxime (non-meningitis)	≤1	2	≥4	≤2	—	—
Cefpodoxime	≤0.5	1	≥2	≤2	—	—
Ceftibuten	—	—	—	≤2	—	—
Ceftriaxone (non-meningitis)	≤1	2	≥4	≤2	—	—
Cefuroxime^b^	≤1	2	≥4	≤4	8	≥16
Clarithromycin	≤0.25	0.5	≥1	≤8	16	≥32
Doxycycline	≤0.25	0.5	≥1	NT	NT	NT
Erythromycin	≤0.25	0.5	≥1	NT	NT	NT
Levofloxacin	≤2	4	≥8	≤2	—	—
Moxifloxacin	≤1	2	≥4	≤1	—	—
Penicillin (2.4 g, 2 MU × 4–6 IV)	≤2	4	≥8	NT	NT	NT
Penicillin (oral)	≤0.06	0.12–1	≥2	NT	NT	NT
Tetracycline	≤1	2	≥4	≤2	4	≥8
Trimethoprim/sulfamethoxazole^c^	≤0.5	1–2	≥4	≤0.5	1–2	≥4

—, not applicable; I, intermediate; NT, not tested; R, resistant; S, susceptible.

^a^Amoxicillin/clavulanic acid was tested at a 2:1 amoxicillin to clavulanic acid ratio; breakpoints are expressed as the amoxicillin component.

^b^Breakpoints used are for cefuroxime axetil (oral).

^c^Trimethoprim/sulfamethoxazole was tested at a 1:19 trimethoprim to sulfamethoxazole ratio; breakpoints are expressed as the trimethoprim component.

**Table 2.** EUCAST (dose-specific) MIC breakpoints (mg/L) used for *S. pneumoniae* and *H. influenzae* isolates

	*S. pneumoniae*		*H. influenzae*	
Antimicrobial^a^	S	R	S	R
Amoxicillin (0.5 g × 3 oral)	≤0.5	>1	≤0.001	>2
Amoxicillin (0.75–1 g × 3 oral)	≤1	>1	≤2	>2
Amoxicillin/clavulanic acid (0.5 g/0.125 g × 3 oral)^b^	≤0.5	>1	≤0.001	>2
Amoxicillin/clavulanic acid (0.875 g/0.125 g × 3 oral)^b^	≤1	>1	≤2	>2
Ampicillin	NT	NT	≤1	>1
Azithromycin	≤0.25	>0.5	—	—
Cefaclor	≤0.001	>0.5	—	—
Cefdinir	—	—	—	—
Cefixime	—	—	≤0.12	>0.12
Cefotaxime	≤0.5	>2	≤0.12	>0.12
Cefpodoxime	≤0.25	>0.5	≤0.25	>0.25
Ceftibuten	—	—	≤1	>1
Ceftriaxone (1 g × 1 IV)	≤0.5	>2	≤0.12	>0.12
Ceftriaxone (2 g × 2 IV)	≤2	>2	≤0.12	>0.12
Cefuroxime^c^	≤0.25	>0.5	≤0.001	>1
Clarithromycin (0.25 g × 2 oral)	≤0.25	>0.5	—	—
Clarithromycin (0.5 g × 2 oral)	≤0.5	>0.5	—	—
Doxycycline	≤1	>2	NT	NT
Erythromycin (0.5 g × 2–4 oral or 0.5 g × 2–4 IV)	≤0.25	>0.5	NT	NT
Erythromycin (1 g × 4 oral or 1 g × 4 IV)	≤0.5	>0.5	NT	NT
Levofloxacin (0.5 g × 2 oral or 0.4 g × 2 IV)	≤0.001	>2	≤0.06	>0.06
Levofloxacin (0.75 g × 2 oral or 0.4 g × 3 IV)	≤2	>2	≤0.06	>0.06
Moxifloxacin	≤0.5	>0.5	≤0.12	>0.12
Penicillin (0.6 g 1 MU × 4 IV)	≤0.06	>2	NT	NT
Penicillin (2.4 g, 2 MU × 4–6 IV)	≤2	>2	NT	NT
Tetracycline	≤1	>2	≤2	>2
Trimethoprim/sulfamethoxazole (0.16 g/0.8 g × 2 oral or IV)^d^	≤1	>2	≤0.5	>1
Trimethoprim/sulfamethoxazole (0.24 g/1.2 g × 2 oral or IV)^d^	≤2	>2	≤1	>1

—, not applicable; I, intermediate; NT, not tested; R, resistant; S, susceptible.

^a^Where available, susceptibility was assessed using EUCAST higher dosage [I (susceptible, increased exposure) category] breakpoints.

^b^Amoxicillin/clavulanic acid was tested at a fixed concentration of 2 mg/L; breakpoints are expressed as the amoxicillin component.

^c^Breakpoints used are for cefuroxime axetil (oral).

^d^Trimethoprim/sulfamethoxazole was tested at a 1:19 trimethoprim to sulfamethoxazole ratio; breakpoints are expressed as the trimethoprim component.

**Table 3.** PK/PD MIC breakpoints (mg/L) used for *S. pneumoniae* and *H. influenzae* isolates

	*S. pneumoniae* and *H. influenzae*
Antimicrobial	S only
Amoxicillin (1.5 g/day)^a^	≤2
Amoxicillin (4 g/day)^b^	≤4
Amoxicillin/clavulanic acid^a^ (1.75 g/0.25 g/day adults; 45 mg/6.4 mg/kg/day children)	≤2
Amoxicillin/clavulanic acid^b^ (4 g/0.25 g/day adults; 90 mg/6.4 mg/kg/day children)	≤4
Ampicillin	—
Azithromycin	≤0.12
Cefaclor	≤0.5
Cefdinir	≤0.25
Cefixime	≤1
Cefotaxime	—
Cefpodoxime	≤0.5
Ceftibuten	—
Ceftriaxone	≤1
Cefuroxime^c^	≤1
Clarithromycin	≤0.25
Doxycycline	≤0.25
Erythromycin	≤0.25
Levofloxacin	≤2
Moxifloxacin	≤1
Penicillin	—
Tetracycline	—
Trimethoprim/sulfamethoxazole^d^	≤0.5

—, not applicable; PK/PD, pharmacokinetic/pharmacodynamic; S, susceptible.

^a^Amoxicillin/clavulanic acid for low dose in adults/children.

^b^Amoxicillin/clavulanic acid for high dose in adults/children.

^c^Breakpoints used are for cefuroxime axetil (oral).

^d^Trimethoprim/sulfamethoxazole was tested at a 1:19 trimethoprim to sulfamethoxazole ratio; breakpoints are expressed as the trimethoprim component.

### Quality control and data analysis

Quality control strains *S. pneumoniae* ATCC 49619, *H. influenzae* ATCC 49247, *H. influenzae* ATCC 49766 and *E. coli* ATCC 32518 were included on each day of testing. Results of susceptibility testing were only accepted if the results of the quality control strains were within the published acceptable range. Differences in susceptibility between Italy and Spain were assessed for statistical significance with Fisher's exact test using XLSTAT version 2023.1.1.1399 (Lumivero, Denver, CO, USA). A P < 0.05 was considered statistically significant. A similar statistical analysis was performed to compare antibiotic susceptibility (using CLSI criteria) by penicillin susceptibility (*S. pneumoniae* only).

## Results

### *S. pneumoniae* isolates

A total of 77 *S. pneumoniae* isolates were collected from Italy between 2018 and 2021. Most isolates came from bronchoalveolar lavage (*n* = 25, 32.5%) or blood (*n* = 21, 27.3%), with the remainder from endotracheal aspirate (*n* = 7, 9.1%), sputum (*n* = 6, 7.8%), middle ear (*n* = 1, 1.3%), sinus (*n* = 1, 1.3%) and unidentified specimens (*n* = 16, 20.8%). Most isolates (*n* = 41, 53.2%) came from adolescents and adults (aged 13–64 years); 28 (36.4%) were from elderly (aged ≥65 years) and 8 (10.4%) were from paediatric patients (aged ≤12 years).

In total, 176 *S. pneumoniae* isolates were collected from Spain between 2018 and 2021. Most isolates came from sputum (n = 102, 58.0%), with the remainder from blood (*n* = 21, 11.9%), endotracheal aspirate (*n* = 18, 10.2%), sinuses (*n* = 13, 7.4%), middle ear (*n* = 7, 4.0%), bronchoalveolar lavage (*n* = 6, 3.4%) and unidentified specimens (*n* = 9, 5.1%). The isolates were evenly spread between adolescent and adult (aged 13–64 years; *n* = 62, 35.2%), elderly (aged ≥65 years; *n* = 60, 34.1%) and paediatric patients (aged ≤12 years; *n* = 52, 29.5%). Two isolates (1.1%) were included without patient age provided.

**Figure 1.**
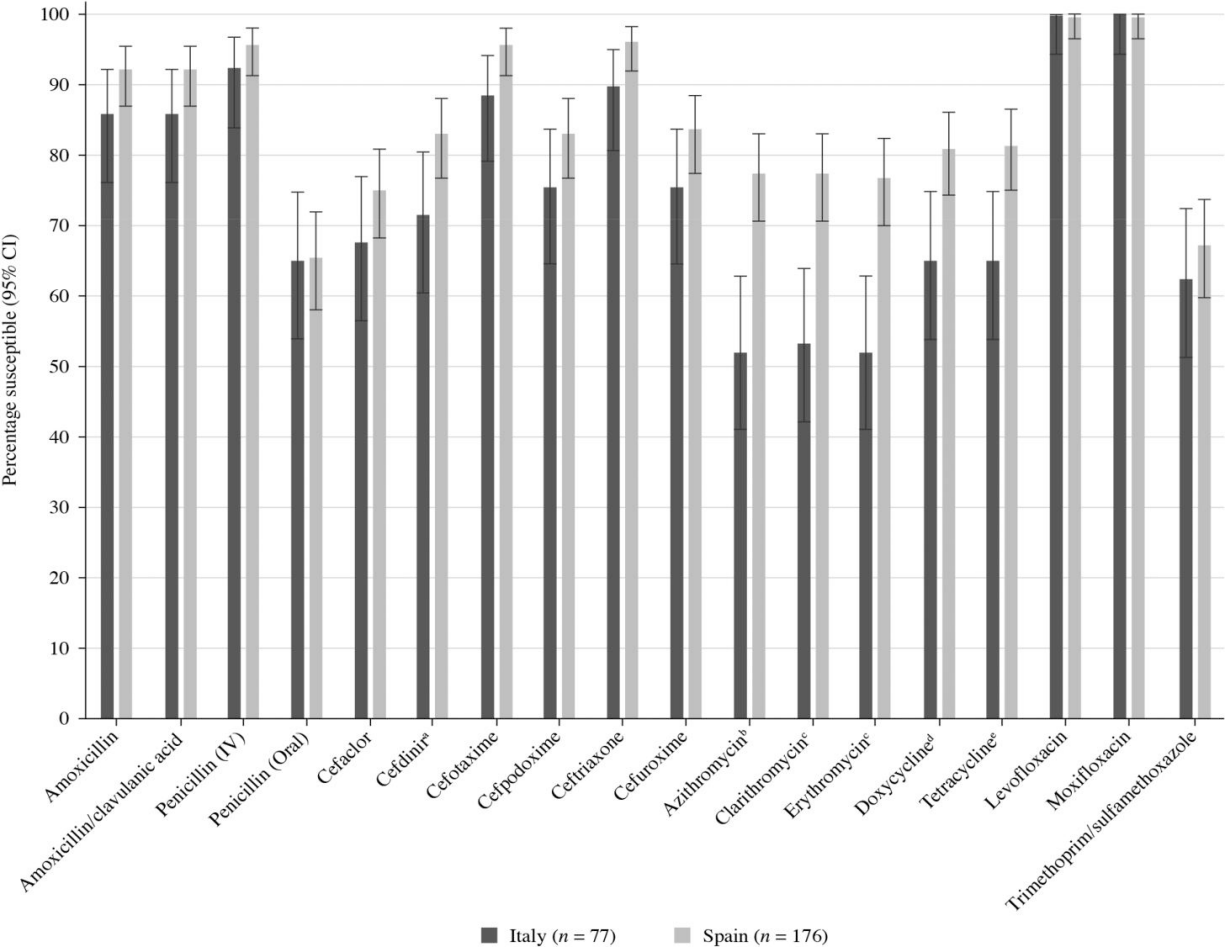
Antibiotic susceptibility rates (with 95% CI) of *S. pneumoniae* isolates from Italy (*n* = 77) and Spain (*n* = 176) based on CLSI breakpoints. Susceptibility was significantly higher in Spain than in Italy: ^a^*P* = 0.04, ^b^*P* < 0.0001, ^c^*P* = 0.0002, ^d^*P* = 0.01 and ^e^*P* = 0.006.

**Figure 2.**
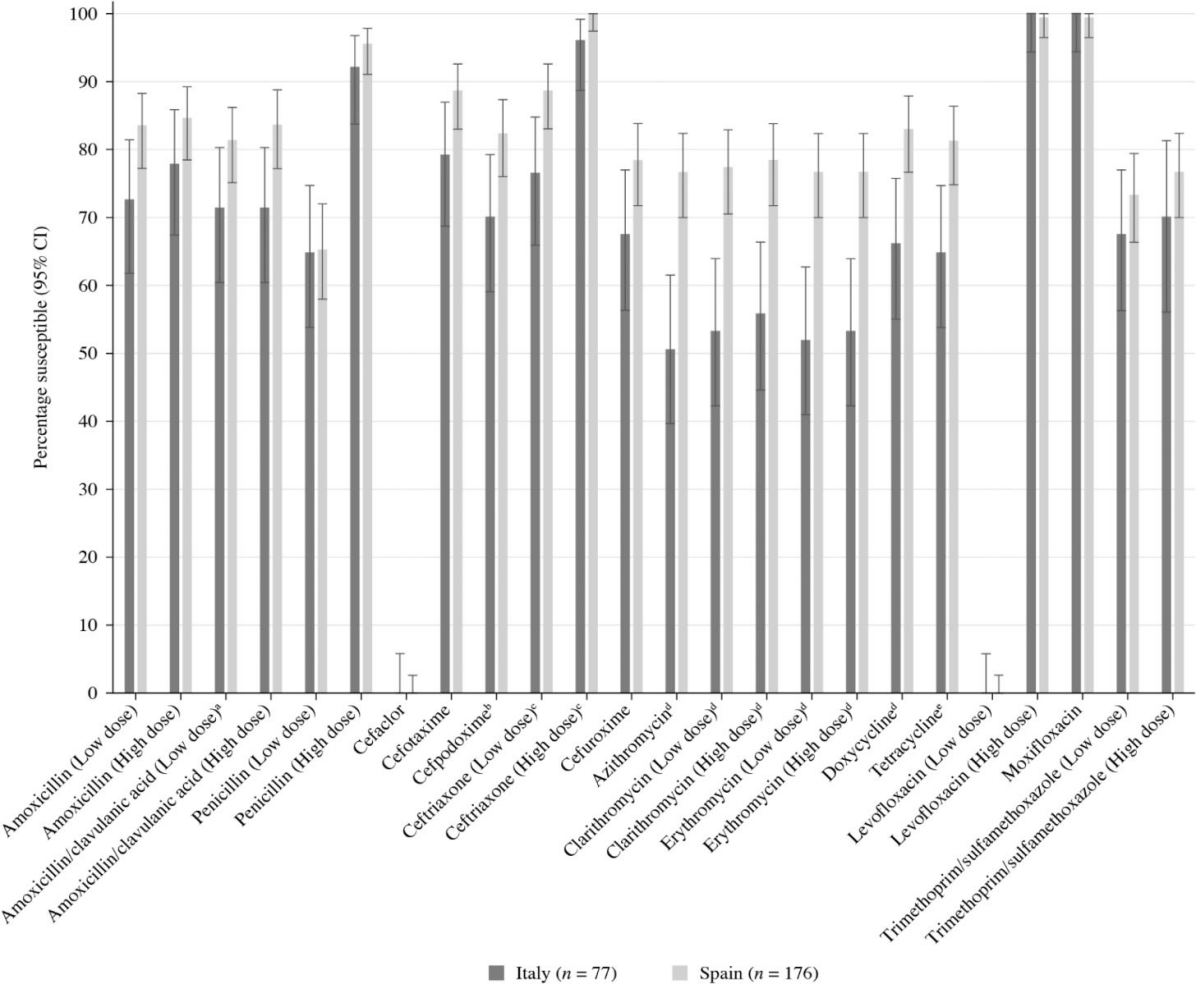
Antibiotic susceptibility rates (with 95% CI) of *S. pneumoniae* from Italy (*n* = 77) and Spain (*n* = 176) based on EUCAST (dose-specific) breakpoints. Susceptibility was significantly higher in Spain than in Italy: ^a^*P* = 0.04, ^b^*P* = 0.03, ^c^*P* = 0.02 or 0.03, ^d^*P* < 0.0001–0.0004 and ^e^*P* = 0.004 or 0.005 (high). CI, confidence interval.

Summary MIC, susceptibility and MIC distribution data for the 77 *S. pneumoniae* isolates from Italy and 176 from Spain are given in Tables 4–9, with MIC distribution data given in Tables S1 and S2 (available as Supplementary data at *JAC* Online), respectively. Comparative susceptibility data for both countries using CLSI and EUCAST breakpoints are shown in Figures 1 and 2.

**Table 4.** MIC and susceptibility data for *S. pneumoniae* isolates (*n* = 77) from Italy using CLSI breakpoints

	MIC (mg/L)			CLSI susceptibility		
Antimicrobial	Range	50%	90%	%S	%I	%R
Amoxicillin	≤0.008–>8	0.03	4	85.7	7.8	6.5
Amoxicillin/clavulanic acid (2:1)	≤0.008–>8	0.03	4	85.7	7.8	6.5
Penicillin (2.4 g, 2 MU × 4–6 IV)	≤0.008–4	0.03	2	92.2	7.8	0
Penicillin (oral)	≤0.008–4	0.03	2	64.9	13	22.1
Cefaclor	0.12–>4	1	>4	67.5	2.6	29.9
Cefdinir	≤0.015–>8	0.12	8	71.4	2.6	26
Cefixime	≤0.25–>16	0.5	>16	—	—	—
Cefotaxime	≤0.008–>4	0.06	2	88.3	6.5	5.2
Cefpodoxime	≤0.015–>4	0.06	>4	75.3	1.3	23.4
Ceftibuten	≤0.5–>16	8	>16	—	—	—
Ceftriaxone	≤0.008–>4	0.06	2	89.6	6.5	3.9
Cefuroxime	≤0.008–>8	0.12	8	75.3	2.6	22.1
Azithromycin	≤0.015–>16	0.25	>16	51.9	0	48.1
Clarithromycin	≤0.015–>16	0.03	>16	53.2	2.6	44.2
Erythromycin	≤0.015–>16	0.06	>16	51.9	1.3	46.8
Doxycycline	≤0.008–>4	0.06	>4	64.9	1.3	33.8
Tetracycline	≤0.03–>4	0.12	>4	64.9	0	35.1
Levofloxacin	≤0.12–2	1	1	100	0	0
Moxifloxacin	≤0.03–0.12	0.12	0.12	100	0	0
Trimethoprim/sulfamethoxazole	≤0.06–8	0.25	8	62.3	7.8	29.9

—, not applicable; I, intermediate; R, resistant; S, susceptible.

**Table 5.** MIC and susceptibility data for *S. pneumoniae* isolates (*n* = 176) from Spain using CLSI breakpoints

	MIC (mg/L)			CLSI susceptibility		
Antimicrobial	Range	50%	90%	%S	%I	%R
Amoxicillin	≤0.008–8	0.03	2	92.0	3.4	4.5
Amoxicillin/clavulanic acid (2:1)	≤0.008–8	0.03	2	92.0	3.4	4.5
Penicillin (2.4 g, 2 MU × 4–6 IV)	≤0.008–4	0.02	2	95.5	4.5	0
Penicillin (oral)	≤0.008–4	0.02	2	65.3	23.3	11.4
Cefaclor	0.03–>4	0.5	>4	75.0	5.1	19.9
Cefdinir	0.03–>8	0.06	4	83.0	0	17.0
Cefixime	≤0.25–>16	≤0.25	16	—	—	—
Cefotaxime	≤0.008–2	0.03	1	95.5	4.5	0
Cefpodoxime	≤0.015–>4	0.03	2	83.0	2.8	14.2
Ceftibuten	1–>16	4	>16	—	—	—
Ceftriaxone	≤0.008–2	0.03	1	96.0	4.0	0
Cefuroxime	≤0.008–8	0.03	4	83.5	5.1	11.4
Azithromycin	≤0.015–>16	0.06	>16	77.3	1.1	21.6
Clarithromycin	≤0.015–>16	≤0.015	>16	77.3	1.1	21.6
Erythromycin	≤0.015–>16	0.03	>16	76.7	0	23.3
Doxycycline	0.015–>4	0.06	4	80.7	1.1	18.2
Tetracycline	0.06–>4	0.12	>4	81.2	1.1	17.6
Levofloxacin	0.25–>8	1	1	99.4	0	0.6
Moxifloxacin	≤0.03–4	0.12	0.12	99.4	0	0.6
Trimethoprim/sulfamethoxazole	≤0.06–>8	0.25	8	67.0	9.7	23.3

—, not applicable; I, intermediate; R, resistant; S, susceptible.

**Table 6.** MIC and susceptibility data for *S. pneumoniae* isolates (*n* = 77) from Italy using EUCAST (dose-specific) breakpoints

	MIC (mg/L)			EUCAST susceptibility		
Antimicrobial	Range	50%	90%	%S	%I	%R
Amoxicillin (0.5 g × 3 oral)	≤0.008–>8	0.03	4	72.7	5.2	22.1
Amoxicillin (0.75–1 g × 3 oral)	≤0.008–>8	0.03	4	77.9	—	22.1
Amoxicillin/clavulanic acid (0.5 g/0.125 g × 3 oral)	≤0.008–>8	0.06	>8	71.4	0	28.6
Amoxicillin/clavulanic acid (0.875 g/0.125 g × 3 oral)	≤0.008–>8	0.06	>8	71.4	—	28.6
Penicillin (0.6 g 1 MU × 4 IV)	≤0.008–4	0.03	2	64.9	27.3	7.8
Penicillin (2.4 g, 2 MU × 4–6 IV)	≤0.008–4	0.03	2	92.2	—	7.8
Cefaclor	0.12–>4	1	>4	—	48.1	51.9
Cefdinir	≤0.015–>8	0.12	8	—	—	—
Cefixime	≤0.25–>16	0.5	>16	—	—	—
Cefotaxime	≤0.008–>4	0.06	2	79.2	15.6	5.2
Cefpodoxime	≤0.015–>4	0.06	>4	70.1	5.2	24.7
Ceftibuten	≤0.5–>16	8	>16	—	—	—
Ceftriaxone (1 g × 1 IV)	≤0.008–>4	0.06	2	76.6	19.5	3.9
Ceftriaxone (2 g × 2 IV)	≤0.008–>4	0.06	2	96.1	—	3.9
Cefuroxime	≤0.008–>8	0.12	8	67.5	5.2	27.3
Azithromycin	≤0.015–>16	0.25	>16	50.6	1.3	48.1
Clarithromycin (0.25 g × 2 oral)	≤0.015–>16	0.03	>16	53.2	2.6	44.2
Clarithromycin (0.5 g × 2 oral)	≤0.015–>16	0.03	>16	55.8	—	44.2
Erythromycin (0.5 g × 2–4 oral or 0.5 g × 2–4 IV)	≤0.015–>16	0.06	>16	51.9	1.3	46.8
Erythromycin (1 g × 4 oral or 1 g × 4 IV)	≤0.015–>16	0.06	>16	53.2	—	46.8
Doxycycline	≤0.008–>4	0.06	>4	66.2	3.9	29.9
Tetracycline	≤0.03–>4	0.12	>4	64.9	0	35.1
Levofloxacin (0.5 g × 2 oral or 0.4 g × 2 IV)	≤0.12–2	1	1	—	100	0
Levofloxacin (0.75 g × 2 oral or 0.4 g × 3 IV)	≤0.12–2	1	1	100	—	0
Moxifloxacin	≤0.03–0.12	0.12	0.12	100	0	0
Trimethoprim/sulfamethoxazole (0.16 g/0.8 g × 2 oral or IV)	≤0.06–8	0.25	8	67.5	2.6	29.9
Trimethoprim/sulfamethoxazole (0.24 g/1.2 g × 2 oral or IV)	≤0.06–8	0.25	8	70.1	—	29.9

—, not applicable; I, intermediate; R, resistant; S, susceptible.

**Table 7.** MIC and susceptibility data for *S. pneumoniae* isolates (*n* = 176) from Spain using EUCAST (dose-specific) breakpoints

	MIC (mg/L)			EUCAST susceptibility		
Antimicrobial	Range	50%	90%	%S	%I	%R
Amoxicillin (0.5 g × 3 oral)	≤0.008–8	0.03	2	83.5	1.1	15.3
Amoxicillin (0.75–1 g × 3 oral)	≤0.008–8	0.03	2	84.7	—	15.3
Amoxicillin/clavulanic acid (0.5 g/0.125 g × 3 oral)	≤0.008–>8	0.06	8	81.2	2.3	16.5
Amoxicillin/clavulanic acid (0.875 g/0.125 g × 3 oral)	≤0.008–>8	0.06	8	83.5	0	16.5
Penicillin (0.6 g 1 MU × 4 IV)	≤0.008–4	0.015	2	65.3	30.1	4.5
Penicillin (2.4 g, 2 MU × 4–6 IV)	≤0.008–4	0.015	2	95.5	—	4.5
Cefaclor	0.03–>4	0.5	>4	—	59.7	40.3
Cefdinir	0.03–>8	0.06	4	—	—	—
Cefixime	≤0.25–>16	≤0.25	16	—	—	—
Cefotaxime	≤0.008–2	0.03	1	88.6	11.4	0
Cefpodoxime	≤0.015–>4	0.03	2	82.4	0.6	17
Ceftibuten	1–>16	4	>16	—	—	—
Ceftriaxone (1 g × 1 IV)	≤0.008–2	0.03	1	88.6	11.4	0
Ceftriaxone (2 g × 2 IV)	≤0.008–2	0.03	1	100	—	0
Cefuroxime	≤0.008–8	0.03	4	78.4	4	17.6
Azithromycin	≤0.015–>16	0.06	>16	76.7	0.6	22.7
Clarithromycin (0.25 g × 2 oral)	≤0.015–>16	≤0.015	>16	77.3	1.1	21.6
Clarithromycin (0.5 g × 2 oral)	≤0.015–>16	≤0.015	>16	78.4	—	21.6
Erythromycin (0.5 g × 2–4 oral or 0.5 g × 2–4 IV)	≤0.015–>16	0.03	>16	76.7	0	23.3
Erythromycin (1 g × 4 oral or 1 g × 4 IV)	≤0.015–>16	0.03	>16	76.7	—	23.3
Doxycycline	0.015–>4	0.06	4	83	1.7	15.3
Tetracycline	0.06–>4	0.12	>4	81.2	1.1	17.6
Levofloxacin (0.5 g × 2 oral or 0.4 g × 2 IV)	0.25–>8	1	1	—	99.4	0.6
Levofloxacin (0.75 g × 2 oral or 0.4 g × 3 IV)	0.25–>8	1	1	99.4	—	0.6
Moxifloxacin	≤0.03–4	0.12	0.12	99.4	—	0.6
Trimethoprim/sulfamethoxazole (0.16 g/0.8 g × 2 oral or IV)	≤0.06–>8	0.25	8	73.3	3.4	23.3
Trimethoprim/sulfamethoxazole (0.24 g/1.2 g × 2 oral or IV)	≤0.06–>8	0.25	8	76.7	—	23.3

—, not applicable; I, intermediate; R, resistant; S, susceptible.

**Table 8.** Summary MIC and susceptibility data for *S. pneumoniae* (*n* = 77) from Italy using PK/PD breakpoints

	MIC (mg/L)			PK/PD susceptibility
Antimicrobial	Range	50%	90%	%S
Amoxicillin (1.5 g/day)	≤0.008–>8	0.03	4	85.7
Amoxicillin (4 g/day)	≤0.008–>8	0.03	4	93.5
Amoxicillin/clavulanic acid (1.75 g/0.25 g/day adults; 45 mg/6.4 mg/kg/day children)	≤0.008–>8	0.03	4	85.7
Amoxicillin/clavulanic acid (4 g/0.25 g/day adults; 90 mg/6.4 mg/kg/day children)	≤0.008–>8	0.03	4	93.5
Penicillin	≤0.008–4	0.03	2	—
Cefaclor	0.12–>4	1	>4	48.1
Cefdinir	≤0.015–>8	0.12	8	70.1
Cefixime	≤0.25–>16	0.5	>16	63.6
Cefotaxime	≤0.008–>4	0.06	2	—
Cefpodoxime	≤0.015–>4	0.06	>4	75.3
Ceftibuten	≤0.5–>16	8	>16	—
Ceftriaxone	≤0.008–>4	0.06	2	89.6
Cefuroxime	≤0.008–>8	0.12	8	75.3
Azithromycin	≤0.015–>16	0.25	>16	49.4
Clarithromycin	≤0.015–>16	0.03	>16	53.2
Erythromycin	≤0.015–>16	0.06	>16	51.9
Doxycycline	≤0.008–>4	0.06	>4	64.9
Tetracycline	≤0.03–>4	0.12	>4	—
Levofloxacin	≤0.12–2	1	1	100
Moxifloxacin	≤0.03–0.12	0.12	0.12	100
Trimethoprim/sulfamethoxazole	≤0.06–8	0.25	8	62.3

—, not applicable; PK/PD, pharmacokinetic/pharmacodynamic; S, susceptible.

**Table 9.** Summary MIC and susceptibility data for *S. pneumoniae* (*n* = 176) from Spain using PK/PD breakpoints

	MIC (mg/L)			PK/PD susceptibility
Antimicrobial	Range	50%	90%	%S
Amoxicillin (1.5 g/day)	≤0.008–8	0.03	2	92.0
Amoxicillin (4 g/day)	≤0.008–8	0.03	2	95.5
Amoxicillin/clavulanic acid (1.75 g/0.25 g/day adults; 45 mg/6.4 mg/kg/day children)	≤0.008–8	0.03	2	92.0
Amoxicillin/clavulanic acid (4 g/0.25 g/day adults; 90 mg/6.4 mg/kg/day children)	≤0.008–8	0.03	2	95.5
Penicillin	≤0.008–4	0.015	2	—
Cefaclor	0.03–>4	0.5	>4	59.7
Cefdinir	0.03–>8	0.06	4	79.0
Cefixime	≤0.25–>16	≤0.25	16	73.9
Cefotaxime	≤0.008–2	0.03	1	—
Cefpodoxime	≤0.015–>4	0.03	2	83.0
Ceftibuten	1–>16	4	>16	—
Ceftriaxone	≤0.008–2	0.03	1	96.0
Cefuroxime	≤0.008–8	0.03	4	83.5
Azithromycin	≤0.015–>16	0.06	>16	76.7
Clarithromycin	≤0.015–>16	≤0.015	>16	77.3
Erythromycin	≤0.015–>16	0.03	>16	76.7
Doxycycline	0.015–>4	0.06	4	80.7
Tetracycline	0.06–>4	0.12	>4	—
Levofloxacin	0.25–>8	1	1	99.4
Moxifloxacin	≤0.03–4	0.12	0.12	99.4
Trimethoprim/sulfamethoxazole	≤0.06–>8	0.25	8	67.0

—, not applicable; PK/PD, pharmacokinetic/pharmacodynamic; S, susceptible.

### *S. pneumoniae* susceptibility

Antibiotic susceptibility by CLSI and EUCAST breakpoints was higher in Spain than in Italy, except for fluoroquinolones. However, this difference was only statistically significant for cefdinir, macrolides and tetracyclines by CLSI breakpoints (Figure 1) and high-dose (0.875 g/0.125 g × 3 oral) amoxicillin/clavulanic acid, cefpodoxime, ceftriaxone, macrolides and tetracyclines by EUCAST breakpoints (Figure 2). Approximately 65% of pneumococci collected in Italy (*n* = 50) and Spain (*n* = 115) were penicillin-susceptible (PSSP) when CLSI oral or EUCAST low-dose IV breakpoints were applied. However, susceptibility to penicillin with EUCAST high-dose and CLSI IV breakpoints increased to 92.2%–95.5%. When following CLSI breakpoints, amoxicillin, amoxicillin/clavulanic acid and the third-generation cephalosporins ceftriaxone and cefotaxime showed similar activity, with susceptibility ranging from 85.7% to 89.6% in Italy and from 92.0% to 96.0% in Spain. Between 83.0% and 83.5%, susceptibility was observed to cefdinir, cefpodoxime and cefuroxime in Spain but susceptibility to these agents in Italy was between 71.4% and 75.3%. The second-generation cephalosporin cefaclor was less active according to CLSI breakpoints in both countries (*n* = 52, 67.5% susceptible in Italy and *n* = 132, 75.0% in Spain). EUCAST breakpoints for amoxicillin and amoxicillin/clavulanic acid are lower than CLSI breakpoints, showing reduced susceptibility compared with that obtained by CLSI breakpoints (71.4%–77.9% in Italy and 81.2%–84.7% in Spain), even if higher-dose breakpoints were used. However, high-dose PK/PD breakpoints for amoxicillin (4 g/day) and amoxicillin/clavulanic acid (4 g/0.25 g/day) increased susceptibility to 93.5% in Italy and 95.5% in Spain. Cephalosporin susceptibility by EUCAST breakpoints is generally lower than that observed with CLSI breakpoints, especially for cefaclor, where no susceptible isolates were observed in either country. However, EUCAST high-dose ceftriaxone retained high susceptibility (96.1% in Italy and 100% in Spain). Cephalosporin susceptibility by PK/PD breakpoints was similar to that by CLSI, with the exception of cefaclor, where 48.1% susceptibility was observed in Italy and 59.7% in Spain. Susceptibility to macrolides (azithromycin, clarithromycin and erythromycin) and tetracyclines (doxycycline and tetracycline) in Italy ranged from 49.4% to 66.2% using CLSI, EUCAST or PK/PD breakpoints. The susceptibility of isolates from Spain to these antibiotics was significantly higher (76.7%–83.0%; *P* < 0.0001–0.002 for macrolides and P = 0.006–0.01 for tetracyclines) using CLSI breakpoints. Similarly, trimethoprim/sulfamethoxazole susceptibility by CLSI, EUCAST or PK/PD breakpoints was lower in Italy (62.3%–70.1% susceptibility) than in Spain (67.0%–76.7%); however, this difference was not statistically significant. Fluoroquinolone susceptibility was ≥99.4% in pneumococci from both countries using CLSI, EUCAST or PK/PD breakpoints, but only if EUCAST high-dose breakpoints were used for levofloxacin (0% susceptible at theoretical off-scale low-dose breakpoint). Only one isolate (from Spain) was resistant to levofloxacin and moxifloxacin by CLSI or EUCAST breakpoints (Tables 5, 7 and S2 and Figures 1 and 2).

### Susceptibility of *S. pneumoniae* by penicillin resistance phenotype

**Figure 3.**
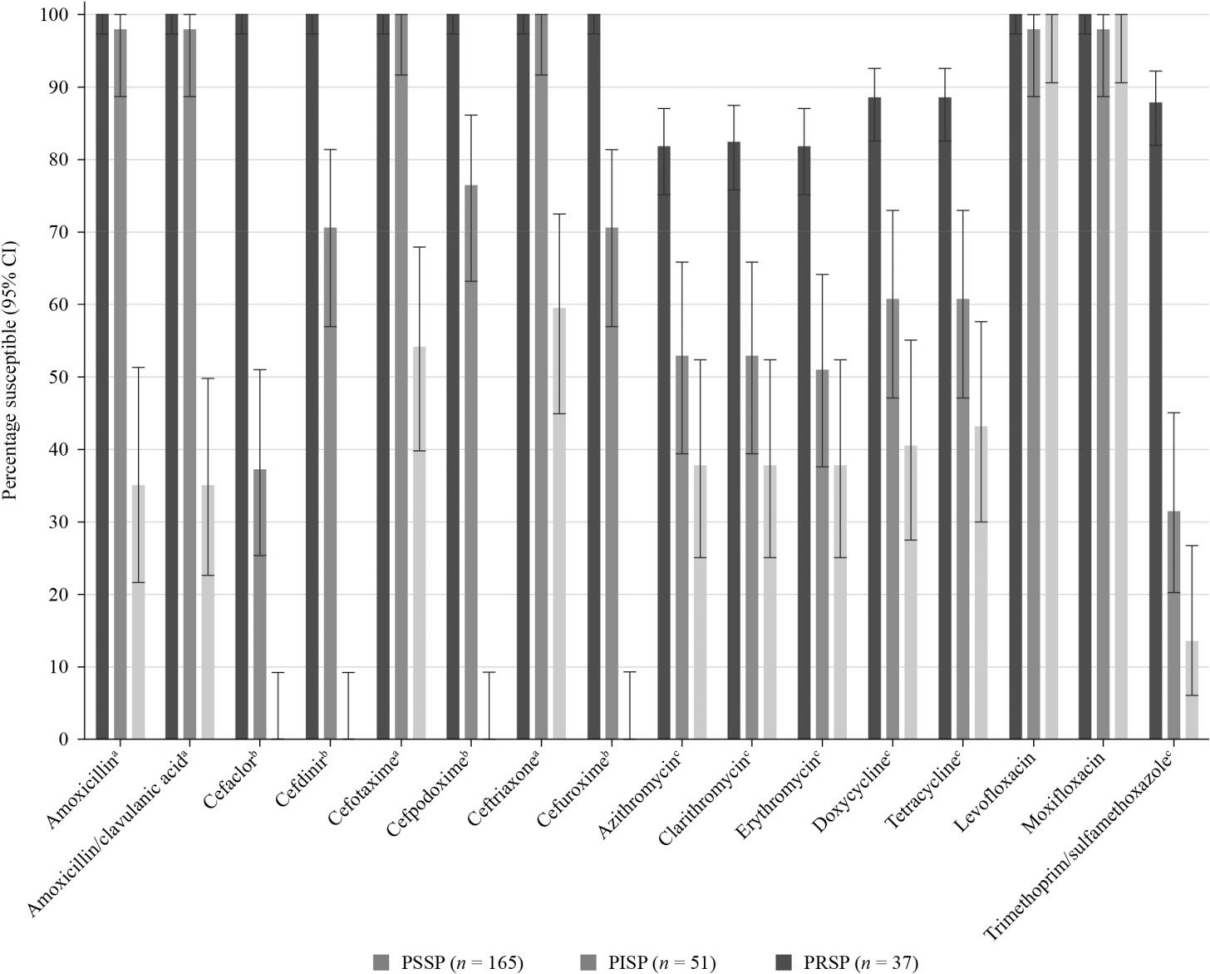
Susceptibility rates (with 95% CI) based on CLSI breakpoints for antibiotics against PSSP, PISP and PRSP from Italy and Spain combined. Penicillin susceptibility categories are based on oral penicillin CLSI breakpoints. ^a^Susceptibility was significantly higher among PSSP and PISP isolates than PRSP isolates (*P* < 0.0001). ^b^Susceptibility was significantly higher among PSSP than PRSP isolates (*P* *<* 0.0001), significantly higher among PISP than PRSP isolates (*P* = 0.006) and significantly higher among PSSP than PISP isolates (*P* < 0.0001). ^c^Susceptibility was significantly higher among PSSP than PISP or PRSP isolates (*P* < 0.0001). CI, confidence interval; PISP, penicillin-intermediate *S. pneumoniae*; PRSP, penicillin-resistant *S. pneumoniae*; PSSP, penicillin-susceptible *S. pneumoniae*.

An analysis of the activity of antimicrobials against pneumococci from Italy and Spain combined based on susceptibility to penicillin (CLSI oral breakpoints) was performed (Figure 3). Of the 253 combined *S. pneumoniae* isolates collected in both countries, 165 (65.2%) were PSSP, 51 (20.2%) were penicillin-intermediate (PISP) and 37 (14.6%) were penicillin-resistant (PRSP) according to CLSI oral breakpoints. PSSP isolates were ≥81.8% susceptible to all antibiotics tested. PSSP isolates showed significantly higher susceptibility rates than PRSP isolates for all antibiotics (*P* < 0.0001), except for the fluoroquinolones, which showed ≥98.0% susceptibility irrespective of penicillin category. PSSP isolates also had significantly higher susceptibility than PISP isolates to cefaclor, cefdinir, cefpodoxime, cefuroxime, macrolides, tetracyclines and trimethoprim/sulfamethoxazole (*P* < 0.0001). Susceptibility rates of PISP isolates to the remaining antibiotics (amoxicillin, amoxicillin/clavulanic acid, cefotaxime, ceftriaxone and fluoroquinolones) were all ≥98.0%. Susceptibility rates of 0%–59.5% were observed for PRSP isolates to all antibiotics, except for levofloxacin and moxifloxacin (100% susceptible).

### *H. influenzae* isolates

In total, 249 *H. influenzae* isolates were collected from Italy during 2018–21. Most isolates originated from bronchoalveolar lavage (*n* = 95, 38.2%) followed by sputum (*n* = 42, 16.9%), endotracheal aspirate (*n* = 28, 11.2%), blood (*n* = 12, 4.8%), sinuses (*n* = 4, 1.6%), middle ear (*n* = 1, 0.4%) and unidentified specimens (*n* = 67, 26.9%). Almost half of these isolates (*n* = 122, 49.0%) came from adolescent and adult patients (aged 13–64 years); 97 isolates (39.0%) were from elderly patients (aged ≥65 years) and 30 isolates (12.0%) from paediatric patients (aged ≤12 years).

**Figure 4.**
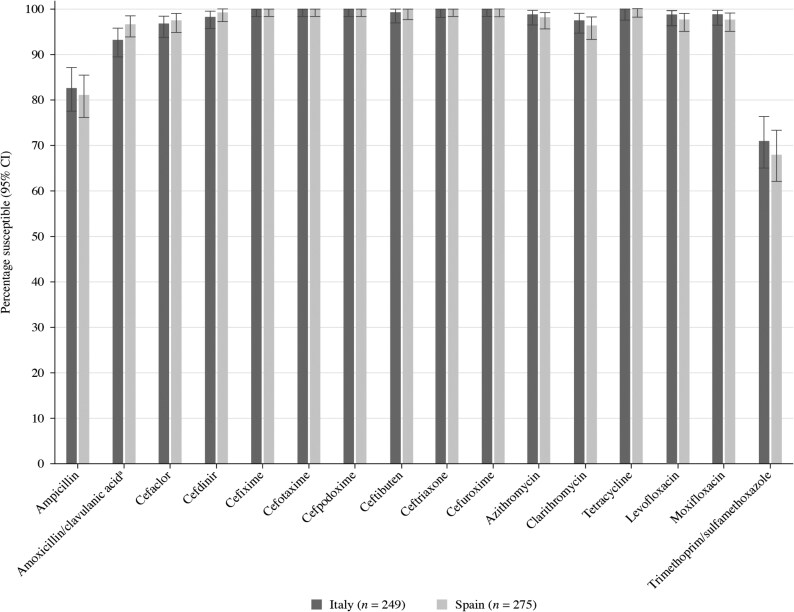
Antibiotic susceptibility rates of *H. influenzae* isolates from Italy (*n* = 249) and Spain (*n* = 275) based on CLSI breakpoints. CI, confidence interval.

**Figure 5.**
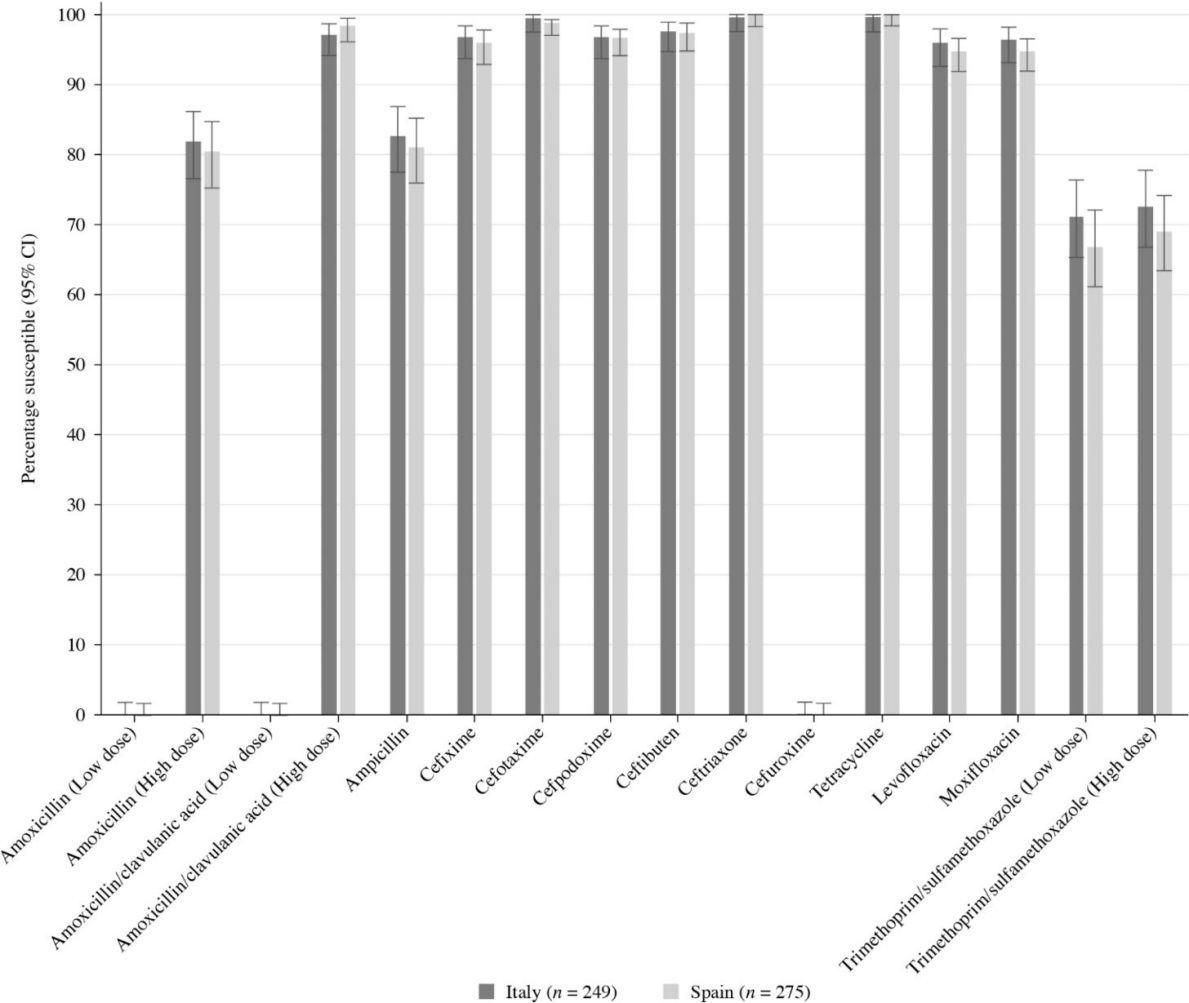
Antibiotic susceptibility rates of *H. influenzae* from Italy (*n* = 249) and Spain (*n* = 275) based on EUCAST (dose-specific) breakpoints. CI, confidence interval.

A total of 275 *H. influenzae* isolates were collected from Spain during 2018–21. Most of these isolates originated from sputum (*n* = 170, 61.8%). The remainder were from endotracheal aspirate (*n* = 35, 12.7%), bronchoalveolar lavage (*n* = 24, 8.7%), sinuses (*n* = 17, 6.2%), middle ear (*n* = 15, 5.5%), blood (*n* = 9, 3.3%) and unidentified specimens (*n* = 5, 1.8%). The isolates were evenly spread between elderly (aged ≥65 years; *n* = 97, 35.3%), adolescent and adult (aged 13–64 years; *n* = 91, 33.1%) and paediatric patients (aged ≤12 years; *n* = 87, 31.6%). Summary MIC, susceptibility and MIC distribution data for *H. influenzae* isolates are given in Tables 10–15 and S3 and S4 and shown in Figures 4 and 5.

**Table 10.** MIC and susceptibility data for *H. influenzae* isolates (*n* = 249) from Italy using CLSI breakpoints

	MIC (mg/L)			CLSI susceptibility		
Antimicrobial	Range	50%	90%	%S	%I	%R
Amoxicillin	≤0.03–128	0.5	16	—	—	—
Ampicillin	≤0.03–>128	0.25	16	82.7	3.6	13.7
Amoxicillin/clavulanic acid (2:1)	≤0.03–8	0.5	2	93.2	5.2	1.6
Cefaclor	≤0.25–32	2	8	96.8	0.8	2.4
Cefdinir	≤0.06–2	0.25	1	98.4	—	—
Cefixime	≤0.008–1	0.03	0.06	100	—	—
Cefotaxime	≤0.002–0.5	0.015	0.03	100	—	—
Cefpodoxime	≤0.015–2	0.06	0.25	100	—	—
Ceftibuten	≤0.008–>4	0.06	0.25	99.2	—	—
Ceftriaxone	≤0.001–0.25	0.004	0.015	100	—	—
Cefuroxime	≤0.03–4	0.5	2	100	0	0
Azithromycin	≤0.12–>8	1	2	98.8	—	—
Clarithromycin	≤0.25–>32	4	8	97.6	0.8	1.6
Tetracycline	≤0.004–>8	0.25	0.5	99.6	0	0.4
Levofloxacin	≤0.004–>8	0.015	0.03	98.8	—	—
Moxifloxacin	≤0.004–>8	0.015	0.03	98.8	—	—
Trimethoprim/sulfamethoxazole	≤0.008–>8	0.12	8	71.1	4.4	24.5

—, not applicable; I, intermediate; R, resistant; S, susceptible.

**Table 11.** MIC and susceptibility data for *H. influenzae* isolates (*n* = 275) from Spain using CLSI breakpoints

	MIC (mg/L)			CLSI susceptibility		
Antimicrobial	Range	50%	90%	%S	%I	%R
Amoxicillin	≤0.03–>128	0.5	32	—	—	—
Ampicillin	≤0.03–>128	0.25	32	81.1	1.1	17.8
Amoxicillin/clavulanic acid (2:1)	≤0.03–16	0.5	2	96.7	2.5	0.7
Cefaclor	≤0.25–>32	2	4	97.5	2.2	0.4
Cefdinir	≤0.06–2	0.25	0.5	99.3	—	—
Cefixime	≤0.008–1	0.03	0.06	100	—	—
Cefotaxime	≤0.002–0.25	0.015	0.03	100	—	—
Cefpodoxime	≤0.015–0.5	0.06	0.25	100	—	—
Ceftibuten	≤0.008–>4	0.06	0.25	99.6	—	—
Ceftriaxone	≤0.001–0.06	0.004	0.015	100	—	—
Cefuroxime	≤0.03–4	0.5	2	100	0	0
Azithromycin	≤0.12–>8	1	2	98.2	—	—
Clarithromycin	≤0.25–>32	4	8	96.4	1.5	2.2
Tetracycline	≤0.12–0.5	0.25	0.5	100	0	0
Levofloxacin	≤0.004–>8	0.015	0.03	97.8	—	—
Moxifloxacin	≤0.004–>8	0.015	0.03	97.8	—	—
Trimethoprim/sulfamethoxazole	≤0.008–>8	0.12	8	68.0	5.8	26.2

—, not applicable; I, intermediate; R, resistant; S, susceptible.

**Table 12.** MIC and susceptibility data for *H. influenzae* isolates (*n* = 249) from Italy using EUCAST (dose-specific) breakpoints

	MIC (mg/L)			EUCAST susceptibility		
Antimicrobial	Range	50%	90%	%S	%I	%R
Amoxicillin (0.5 g × 3 oral)	≤0.03–128	0.5	16	—	81.9	18.1
Amoxicillin (0.75–1 g × 3 oral)	≤0.03–128	0.5	16	81.9	—	18.1
Amoxicillin/clavulanic acid (0.5 g/0.125 g × 3 oral)	≤0.03–8	0.5	2	—	97.2	2.8
Amoxicillin/clavulanic acid (0.875 g/0.125 g × 3 oral)	≤0.03–8	0.5	2	97.2	—	2.8
Ampicillin	≤0.03–>128	0.25	16	82.7	0	17.3
Cefaclor	≤0.25–32	2	8	—	—	—
Cefdinir	≤0.06–2	0.25	1	—	—	—
Cefixime	≤0.008–1	0.03	0.06	96.8	—	3.2
Cefotaxime	≤0.002–0.5	0.015	0.03	99.6	—	0.4
Cefpodoxime	≤0.015–2	0.06	0.25	96.8	—	3.2
Ceftibuten	≤0.008–>4	0.06	0.25	97.6	—	2.4
Ceftriaxone	≤0.001–0.25	0.004	0.015	99.6	—	0.4
Cefuroxime	≤0.03–4	0.5	2	—	82.3	17.7
Azithromycin	≤0.12–>8	1	2	—	—	—
Clarithromycin	≤0.25–>32	4	8	—	—	—
Tetracycline	≤0.12–8	0.25	0.5	99.6	—	0.4
Levofloxacin	≤0.004–>8	0.015	0.03	96.0	—	4.0
Moxifloxacin	≤0.004–>8	0.015	0.03	96.4	—	3.6
Trimethoprim/sulfamethoxazole (0.16 g/0.8 g × 2 oral or IV)	≤0.008–>8	0.12	8	71.1	1.6	27.3
Trimethoprim/sulfamethoxazole (0.24 g/1.2 g × 2 oral or IV)	≤0.008–>8	0.12	8	72.7	—	27.3

—, not applicable; I, intermediate; R, resistant; S, susceptible.

**Table 13.** MIC and susceptibility data for *H. influenzae* isolates (*n* = 275) from Spain using EUCAST (dose-specific) breakpoints

	MIC (mg/L)			EUCAST susceptibility		
Antimicrobial	Range	50%	90%	%S	%I	%R
Amoxicillin (0.5 g × 3 oral)	≤0.03–>128	0.5	32	—	80.7	19.3
Amoxicillin (0.75–1 g × 3 oral)	≤0.03–>128	0.5	32	80.7	—	19.3
Amoxicillin/clavulanic acid (0.5 g/0.125 g × 3 oral)	≤0.03–4	0.5	1	—	98.5	1.5
Amoxicillin/clavulanic acid (0.875 g/0.125 g × 3 oral)	≤0.03–4	0.5	1	98.5	—	1.5
Ampicillin	≤0.03–>128	0.25	32	81.1	—	18.9
Cefaclor	≤0.25–>32	2	4	—	—	—
Cefdinir	≤0.06–>4	0.25	0.5	—	—	—
Cefixime	≤0.008–1	0.03	0.06	96.7	—	3.3
Cefotaxime	≤0.002–0.25	0.015	0.03	99.6	—	0.4
Cefpodoxime	≤0.015–0.5	0.06	0.25	97.8	—	2.2
Ceftibuten	≤0.008–>4	0.06	0.25	98.2	—	1.8
Ceftriaxone	≤0.001–0.06	0.004	0.015	100	—	0
Cefuroxime	≤0.03–4	0.5	2	—	87.3	12.7
Azithromycin	≤0.12–>8	1	2	—	—	—
Clarithromycin	≤0.25–>32	4	8	—	—	—
Tetracycline	≤0.12–0.5	0.25	0.5	100	—	0
Levofloxacin	≤0.004–>8	0.015	0.03	96.7	—	3.3
Moxifloxacin	≤0.004–>8	0.015	0.03	96.7	—	3.3
Trimethoprim/sulfamethoxazole (0.16 g/0.8 g × 2 oral or IV)	≤0.008–>8	0.12	8	68.0	1.8	30.2
Trimethoprim/sulfamethoxazole (0.24 g/1.2 g × 2 oral or IV)	≤0.008–>8	0.12	8	69.8	—	30.2

—, not applicable; I, intermediate; R, resistant; S, susceptible.

**Table 14.** Summary MIC and susceptibility data for *H. influenzae* (*n* = 249) from Italy using PK/PD breakpoints

	MIC (mg/L)			PK/PD susceptibility
Antimicrobial	Range	50%	90%	%S
Amoxicillin (1.5 g/day)	≤0.03–128	0.5	16	81.9
Amoxicillin (4 g/day)	≤0.03–128	0.5	16	85.5
Amoxicillin/clavulanic acid (1.75 g/0.25 g/day adults; 45 mg/6.4 mg/kg/day children)	≤0.03–8	0.5	2	93.2
Amoxicillin/clavulanic acid (4 g/0.25 g/day adults; 90 mg/6.4 mg/kg/day children)	≤0.03–8	0.5	2	98.4
Ampicillin	≤0.03–>128	0.25	16	—
Cefaclor	≤0.25–32	2	8	6.4
Cefdinir	≤0.06–2	0.25	1	71.5
Cefixime	≤0.008–1	0.03	0.06	100
Cefotaxime	≤0.002–0.5	0.015	0.03	—
Cefpodoxime	≤0.015–2	0.06	0.25	97.2
Ceftibuten	≤0.008–>4	0.06	0.25	—
Ceftriaxone	≤0.001–0.25	0.004	0.015	100
Cefuroxime	≤0.03–4	0.5	2	82.3
Azithromycin	≤0.12–>8	1	2	2.4
Clarithromycin	≤0.25–>32	4	8	0.4
Tetracycline	≤0.12–8	0.25	0.5	—
Levofloxacin	≤0.004–>8	0.015	0.03	98.8
Moxifloxacin	≤0.004–>8	0.015	0.03	98.8
Trimethoprim/sulfamethoxazole	≤0.008–>8	0.12	8	71.1

—, not applicable; PK/PD, pharmacokinetic/pharmacodynamic; S, susceptible.

**Table 15.** Summary MIC and susceptibility data for *H. influenzae* (*n* = 275) from Spain using PK/PD breakpoints

	MIC (mg/L)			PK/PD susceptibility
Antimicrobial	Range	50%	90%	%S
Amoxicillin (1.5 g/day)	≤0.03–>128	0.5	32	80.7
Amoxicillin (4 g/day)	≤0.03–>128	0.5	32	84.0
Amoxicillin/clavulanic acid (1.75 g/0.25 g/day adults; 45 mg/6.4 mg/kg/day children)	≤0.03–4	0.5	2	96.7
Amoxicillin/clavulanic acid (4 g/0.25 g/day adults; 90 mg/6.4 mg/kg/day children)	≤0.03–4	0.5	2	99.3
Ampicillin	≤0.03–>128	0.25	32	—
Cefaclor	≤0.25–>32	2	4	6.2
Cefdinir	≤0.06–>4	0.25	0.5	75.6
Cefixime	≤0.008–1	0.03	0.06	100
Cefotaxime	≤0.002–0.5	0.015	0.03	—
Cefpodoxime	≤0.015–2	0.06	0.25	100
Ceftibuten	≤0.008–>4	0.06	0.25	—
Ceftriaxone	≤0.001–0.12	0.004	0.015	100
Cefuroxime	≤0.03–>16	0.5	2	87.3
Azithromycin	≤0.12–>8	1	2	3.3
Clarithromycin	≤0.25–>32	4	8	1.5
Tetracycline	≤0.12–0.5	0.25	0.5	—
Levofloxacin	≤0.004–>8	0.015	0.03	97.8
Moxifloxacin	≤0.004–>8	0.015	0.03	97.8
Trimethoprim/sulfamethoxazole	≤0.008–>8	0.12	8	68.0

—, not applicable; PK/PD, pharmacokinetic/pharmacodynamic; S, susceptible.

### *H. influenzae* susceptibility

Most isolates of *H. influenzae* from Italy (214/249, 85.9%) and Spain (228/275, 82.9%) were β-lactamase negative. Within these populations, 10 isolates from Italy and nine isolates from Spain were β-lactamase negative ampicillin-resistant (BLNAR) by EUCAST breakpoints (ampicillin MIC ≥ 2 mg/L). Following CLSI breakpoints (ampicillin MIC ≥ 4 mg/L), one isolate from Italy and seven from Spain were BLNAR. In keeping with this β-lactamase prevalence and BLNAR status, 82.7% of isolates from Italy and 81.1% of isolates from Spain were susceptible to ampicillin (CLSI or EUCAST breakpoints); however, this difference was not statistically significant. A few isolates (four from Spain and two from Italy) were β-lactamase positive but ampicillin-susceptible, regardless of the breakpoints applied. Amoxicillin breakpoints are not provided by CLSI, but no isolate was susceptible using low-dose EUCAST breakpoints (0.5 g × 3 oral) amoxicillin. However, at EUCAST high-dose (0.75–1 g × 3 oral) or PK/PD low-dose (1.5 g/day), susceptibility was 81.9% in Italy and 80.7% in Spain. PK/PD high dose (4 g/day) increased susceptibility to 85.5% in Italy and 84.0% in Spain. Susceptibility of isolates to amoxicillin/clavulanic acid (2:1) by CLSI breakpoints was 93.2% (*n* = 232) in Italy and 96.7% (*n* = 266) in Spain. Amoxicillin/clavulanic acid (2 mg/L) susceptibility using high-dose (0.875 g/0.125 g × 3 oral) EUCAST breakpoints was 97.2% (*n* = 242) in Italy and 98.5% (*n* = 271) in Spain. Slightly higher susceptibility was observed using high-dose PK/PD breakpoints in both countries. Where breakpoints exist, susceptibility to most other antimicrobials was ≥94.9% by CLSI or EUCAST, with the exception of trimethoprim/sulfamethoxazole, where susceptibility ranged from 68.0% to 72.7% by CLSI, EUCAST and PK/PD breakpoints (Tables 9–15 and S1 and S2 and Figures 4 and 5). PK/PD breakpoints differed for cefaclor where few isolates would be considered susceptible. Lower cefdinir and macrolide susceptibility was also observed by PK/PD compared with CLSI, but this was more in keeping with EUCAST where breakpoints are not given for these agents.

## Discussion

SOAR is an ongoing global surveillance study focusing on the two main CA-RTI pathogens, *S. pneumoniae* and *H. influenzae*, that has monitored numerous countries since 2002, but has investigated Italy and Spain for the first time in 2018–21. The data presented here are an analysis of the antibiotic susceptibility of *S. pneumoniae* and *H. influenzae* isolates collected in Italy and Spain between 2018 and 2021.

Macrolide and tetracycline susceptibility of *S. pneumoniae* in Spain was statistically higher than in Italy when using both CLSI and EUCAST breakpoints. Nevertheless, susceptibility of isolates from Spain to these agents was no greater than 83.0%, indicating that macrolides and tetracyclines are not a good choice for empirical therapy in either country. The susceptibility of pneumococci to trimethoprim/sulfamethoxazole was also low in both countries (≤76.7%). Similarly, the SOAR data from Italy and Spain suggest that oral penicillin or low-dose IV penicillin is also not an appropriate treatment regimen for CA-RTIs, as data using EUCAST low-dose IV or CLSI oral breakpoints showed 64.9% susceptibility in Italy and 65.3% in Spain. Data from the current study support higher-dose IV penicillin as a better therapeutic option, with susceptibility of 92.2% in Italy and 95.5% in Spain. This approach has been recommended in a recent publication on community-acquired infections in Spain.^16^ Susceptibility to some of the other agents was also significantly higher in Spain compared with Italy, including cefdinir using CLSI breakpoints (83.0% versus 71.4%), low-dose amoxicillin/clavulanic acid (81.3% versus 71.4%), cefpodoxime (82.4% versus 70.1%), low-dose ceftriaxone (88.6% versus 76.6%) and high-dose ceftriaxone (100% versus 96.1%) using EUCAST breakpoints. Susceptibility following both guidelines indicated good activity for fluoroquinolones (100% susceptible in Italy and 99.4% in Spain) against *S. pneumoniae*. Furthermore, apart from fluoroquinolones, there was a clear association between low penicillin susceptibility and low susceptibility to other antibiotics.

It is interesting to note that the SENTRY surveillance study data accessible from an online database over the same study period show higher antibiotic susceptibility in Italy compared with Spain.^17^ Our separate statistical analysis using CLSI breakpoints (data not shown) showed a significant difference between Italy and Spain for amoxicillin/clavulanic acid (96.1% versus 85.2%), ceftriaxone (89.6% versus 81.5%), trimethoprim/sulfamethoxazole (77.6% versus 68.4%), oral penicillin (78.1% versus 67.1%) and IV penicillin (96.4% versus 88.5%). However, another dataset from Spain in 2006–07 showed susceptibility data for *S. pneumoniae* that was more in keeping with this current SOAR study than with SENTRY.^18^ This reflects the inherent limitation for all surveillance studies where differences in susceptibility can occur between sites within the same country.

*H. influenzae* from Italy and Spain were mainly β-lactamase negative (85.9% and 82.9%, respectively), with 10 BLNAR isolates from Italy and nine from Spain using EUCAST breakpoints and one from Italy and seven from Spain by CLSI breakpoints. Ampicillin susceptibility was in accordance with β-lactamase prevalence (82.7% in Italy and 81.1% in Spain by EUCAST breakpoints). When comparing the countries, any numerical difference in susceptibility between Italy and Spain was not statistically significant using EUCAST breakpoints or CLSI breakpoints. Susceptibility to amoxicillin/clavulanic acid was high in both countries. Apart from trimethoprim/sulfamethoxazole (71.1% susceptible in Italy and 68.0% susceptible in Spain) and ampicillin (82.7% susceptible in Italy and 81.1% susceptible in Spain), most isolates were highly susceptible to the other antibiotics tested by CLSI breakpoints (≥96.8% in Italy and ≥96.4% in Spain). Some EUCAST breakpoints are much lower than their CLSI counterparts, demonstrating reduced susceptibility as seen with cefuroxime (100% by CLSI versus 82.3%–87.3% by EUCAST increased exposure). Data from the SENTRY surveillance interactive database for *H. influenzae* collected from 2018 to 2021 show similar activity for Italy and Spain, confirming the results of the current study.^18^

The differences in susceptibility between blood and non-blood isolates have been evaluated globally; while the results are not included in this manuscript, they were presented at ESCMID Global 2025.^19,20^ For *S. pneumoniae* and *H. influenzae*, susceptibility rates for most antibiotics were similar between blood and non-blood isolates. Nonetheless, some non-blood isolates showed reduced susceptibility compared with blood isolates, specifically penicillin (oral), trimethoprim/sulfamethoxazole and second-generation cephalosporins (*S. pneumoniae*) and aminopenicillins, trimethoprim/sulfamethoxazole and levofloxacin (*H. influenzae*).

The inclusion of patients who may have received antibiotics before sample collection represents a potential limitation in both the present and previous SOAR studies, as antibiotic pretreatment could have promoted the selection of resistant bacterial strains. However, as a real-world evaluation of antibiotic resistance, the SOAR programme aims to capture the resistance patterns encountered by clinicians globally. Thus, patients with prior antibiotic treatment were not excluded, in line with standard practices in many settings where empirical antibiotic treatment precedes sample collection.

To conclude, antimicrobial susceptibility of *S. pneumoniae* isolates to many antibiotics tested was low in Italy and Spain, with susceptibility > 90% according to both CLSI and EUCAST guidelines observed only with high-dose penicillin and fluoroquinolones. Further, reduced susceptibility was associated with penicillin resistance. Higher antimicrobial susceptibility was seen with *H. influenzae* compared with *S. pneumoniae* in both countries. Continued surveillance of antibiotic susceptibility is required to regularly assess any future changes.

## Supplementary Material

dkaf283_Supplementary_Data

## Transparency declarations

This article forms part of a Supplement sponsored by GSK. D.T. is employed by GSK and holds financial equities in GSK. R.C. has received payment or honoraria for participation in educational programmes sponsored by GSK, Menarini, MSD and Shionogi and has received grants or contracts for research projects or surveillance studies from GSK, MSD, Pfizer and Shionogi. C.P. has received payment or honoraria from MSD, Pfizer and Shionogi and has received support for attending meetings and/or travel from Menarini, Pfizer and Shionogi. M.S., C.V., C.F. and V.S. have nothing to declare. S.H. is employed by IHMA Europe Sàrl. A.M. is employed by GSK and holds financial equities in GSK.

## Supplementary data

Tables S1–S4 are available as Supplementary data at *JAC* Online.

## References

[1] Aliberti S, Dela Cruz CS, Amati F et al Community-acquired pneumonia. Lancet 2021; 398: 906–19. 10.1016/S0140-6736(21)00630-9 34481570

[2] Cillóniz C, Dominedò C, Garcia-Vidal C et al Community-acquired pneumonia as an emergency condition. Curr Opin Crit Care 2018; 24: 531–9. 10.1097/MCC.0000000000000550 30239410

[3] Sijbom M, Büchner FL, Saadah NH et al Trends in antibiotic selection pressure generated in primary care and their association with sentinel antimicrobial resistance patterns in Europe. J Antimicrob Chemother 2023; 78: 1245–52. 10.1093/jac/dkad082 37005341 PMC10154126

[4] Bell BG, Schellevis F, Stobberingh E et al A systematic review and meta-analysis of the effects of antibiotic consumption on antibiotic resistance. BMC Infect Dis 2014; 14:13. 10.1186/1471-2334-14-13 24405683 PMC3897982

[5] Jain S, Self WH, Wunderink RG et al Community-acquired pneumonia requiring hospitalization among U.S. adults. N Engl J Med 2015; 373: 415–27. 10.1056/NEJMoa1500245 26172429 PMC4728150

[6] Gadsby NJ, Russell CD, McHugh MP et al Comprehensive molecular testing for respiratory pathogens in community-acquired pneumonia. Clin Infect Dis 2016; 62: 817–23. 10.1093/cid/civ1214 26747825 PMC4787606

[7] Peyrani P, Mandell L, Torres A et al The burden of community-acquired bacterial pneumonia in the era of antibiotic resistance. Expert Rev Respir Med 2019; 13: 139–52. 10.1080/17476348.2019.1562339 30596308

[8] Heinz E . The return of Pfeiffer’s bacillus: rising incidence of ampicillin resistance in *Haemophilus influenzae*. Microb Genom 2018; 4: e000214. 10.1099/mgen.0.000214 30207515 PMC6202453

[9] World Health Organization . Global antimicrobial resistance and use surveillance system (GLASS). https://www.who.int/initiatives/glass.

[10] Cantón R, Akova M, Langfeld K et al Relevance of the consensus principles for appropriate antibiotic prescribing in 2022. J Antimicrob Chemother 2022; 77(Suppl 1): i2–9. 10.1093/jac/dkac211 36065724 PMC9445850

[11] Torumkuney D, Chaiwarith R, Reechaipichitkul W et al Results from the Survey of Antibiotic Resistance (SOAR) 2012-14 in Thailand, India, South Korea and Singapore. J Antimicrob Chemother 2016; 71(Suppl 1): i3–i19. 10.1093/jac/dkw073 27048580 PMC4890353

[12] CLSI . Methods for Dilution Antimicrobial Susceptibility Tests for Bacteria that Grow Aerobically—Twelfth Edition: M07. https://clsi.org/shop/standards/m07/.

[13] CLSI . Performance Standards for Antimicrobial Susceptibility Testing—Thirty-Fifth Edition: M100. https://clsi.org/shop/standards/m100/.

[14] European Committee on Antimicrobial Susceptibility Testing . Breakpoint tables for interpretation of MICs and zone diameters. Version 12.0, 2022. http://www.eucast.org/fileadmin/src/media/PDFs/EUCAST_files/Breakpoint_tables/v_12.0_Breakpoint_Tables.pdf.

[15] Anon JB, Jacobs MR, Poole MD et al Antimicrobial treatment guidelines for acute bacterial rhinosinusitis. Otolaryngol Head Neck Surg 2004; 130(Suppl 1): 1–45. 10.1016/j.otohns.2003.12.003 14726904 PMC7118847

[16] Llor C, Hoyos Mallecot Y, Moragas A et al New paradigms on antibiotic recommendations for community-acquired infections in Spain. Aten Primaria 2023; 55:102648. 10.1016/j.aprim.2023.102648 37167756 PMC10188543

[17] SENTRY Antimicrobial Surveillance Program . Microbiology Visualization Platform. https://sentry-mvp.jmilabs.com.

[18] Pérez-Trallero E, Martín-Herrero JE, Mazón A et al Antimicrobial resistance among respiratory pathogens in Spain: latest data and changes over 11 years (1996–1997 to 2006–2007). Antimicrob Agents Chemother 2010; 54: 2953–9. 10.1128/AAC.01548-09 20439616 PMC2897302

[19] Manoharan A, Torumkuney D, Morrissey I et al Comparison of antimicrobial susceptibility of *Streptococcus pneumoniae* and *Haemophilus influenzae* from blood and non-blood cultures: analysis from the survey of antibiotic resistance (SOAR) study, 2018–2021. ESCMID Global 2025, Vienna, Austria. Abstract P1200 | 02329.

[20] Manoharan A, Torumkuney D, Morrissey I et al Comparison of antimicrobial susceptibility of *Streptococcus pneumoniae* and *Streptococcus pneumoniae* from blood and non-blood cultures: analysis from the survey of antibiotic resistance (SOAR) study, 2018–2021. Presented at ESCMID Global 2025, Vienna, Austria (Poster 1499).

