# Supplementary material for: Results from the Survey of Antibiotic Resistance (SOAR) 2018–21 in Italy and Spain: data based on CLSI, EUCAST (dose-specific) and pharmacokinetic/pharmacodynamic (PK/PD) breakpoints
Source: J Antimicrob Chemother. 2025 Nov 24;80(Suppl 3):iii2–iii18. doi: 10.1093/jac/dkaf283 (PMC12641132; doi:10.1093/jac/dkaf283)
Supplement: dkaf283_Supplementary_Data [file dkaf283_supplementary_data.docx]

**Results from the Survey of Antibiotic Resistance (SOAR) 2018 – 21 in Italy and Spain: data based on CLSI, EUCAST (dose-specific) and pharmacokinetic/pharmacodynamic (PK/PD) breakpoints**

**Authors:** Didem TORUMKUNEY^1^, Rafael CANTON^2^, Cristina PITART^3^, Maurizio SANGUINETTI^4^, Chiara VISMARA^5^, Claudio FARINA^6^, Vittorio SAMBRI^7^, Stephen HAWSER^8^, Anand MANOHARAN^9*^

**Affiliations:** ^1^GSK, London, UK; ^2^Hospital Universitario Ramon y Cajal and Instituto Ramón y Cajal de Investigación Sanitaria (IRYCIS), Madrid, Spain; and CIBER de Enfermedades Infecciosas (CIBERINFEC), Madrid, Spain; ^3^Department of Microbiology, Hospital Clinic, University of Barcelona, ISGLOBAL, Barcelona, Spain; ^4^Dipartimento di Scienze di Laboratorio e Infettivologiche, Fondazione Policlinico Universitario A. Gemelli IRCCS, Roma, Italy; ^5^Azienda Socio-Sanitaria Territoriale (ASST) Grande Ospedale Metropolitano (GOM) Niguarda, Milano, Italy; ^6^Azienda Socio-Sanitaria Territoriale (ASST) Papa Giovanni XXIII, Bergamo, Italy; ^7^Department of Experimental, Diagnostic and Specialty Medicine-DIMES, Alma Mater Studiorum-University of Bologna, Bologna, Italy; ^8^IHMA Europe Sàrl, Rte. De I’Ile-au-Bois 1A, 1870 Monthey, Switzerland; ^9^Infectious Diseases Medical & Scientific Affairs, GSK, Mumbai, India.

*Corresponding author. E-mail: anand.x.manoharan@gsk.com
**Running title:** Survey of Antibiotic Resistance (SOAR) in Italy and Spain in 2018 – 21

**Supplementary Table 1.** MIC distribution data for *S. pneumoniae* isolates (*n* = 77) from Italy

|  |  | Number of isolates at MIC (mg/L) | | | | | | | | | | | | | | | | | | | | |
| --- | --- | --- | --- | --- | --- | --- | --- | --- | --- | --- | --- | --- | --- | --- | --- | --- | --- | --- | --- | --- | --- | --- |
| Antimicrobial |  | ≤0.008 | ≤0.015 | 0.015 | ≤0.03 | 0.03 | ≤0.06 | 0.06 | ≤0.12 | 0.12 | ≤0.25 | 0.25 | ≤0.5 | 0.5 | 1 | 2 | 4 | >4 | 8 | >8 | 16 | >16 |
| AMX | N | 4 | – | 25 | – | 16 | – | 2 | – | 5 | – | 3 | – | 1 | 4 | 6 | 6 | – | 4 | 1 | – | – |
|  | Cum. % | 5.2 | – | 37.7 | – | 58.4 | – | 61.0 | – | 67.5 | – | 71.4 | – | 72.7 | 77.9 | 85.7 | 93.5 | – | 98.7 | 100 | – | – |
|  | % | 5.2 | – | 32.5 | – | 20.8 | – | 2.6 | – | 6.5 | – | 3.9 | – | 1.3 | 5.2 | 7.8 | 7.8 | – | 5.2 | 1.3 | – | – |
| AMC (2:1) | N | 15 | – | 16 | – | 15 | – | 1 | – | 5 | – | 3 | – | 1 | 3 | 7 | 6 | – | 4 | 1 | – | – |
|  | Cum. % | 19.5 | – | 40.3 | – | 59.7 | – | 61.0 | – | 67.5 | – | 71.4 | – | 72.7 | 76.6 | 85.7 | 93.5 | – | 98.7 | 100 | – | – |
|  | % | 19.5 | – | 20.8 | – | 19.5 | – | 1.3 | – | 6.5 | – | 3.9 | – | 1.3 | 3.9 | 9.1 | 7.8 | – | 5.2 | 1.3 | – | – |
| AMC  [2 mg/L] | N | 16 | – | 2 | – | 9 | – | 20 | – | 1 | – | 5 | – | 2 | – | 1 | 4 | – | 6 | 11 | – | – |
|  | Cum. % | 20.8 | – | 23.4 | – | 35.1 | – | 61.0 | – | 62.3 | – | 68.8 | – | 71.4 | – | 72.7 | 77.9 | – | 85.7 | 100 | – | – |
|  | % | 20.8 | – | 2.6 | – | 11.7 | – | 26.0 | – | 1.3 | – | 6.5 | – | 2.6 | – | 1.3 | 5.2 | – | 7.8 | 14.3 | – | – |
| AZM | N | – | 4 | – | – | 6 | – | 21 | – | 7 | – | 1 | – | 1 | – | 3 | 4 | – | 3 | – | – | 27 |
|  | Cum. % | – | 5.2 | – | – | 13.0 | – | 40.3 | – | 49.4 | – | 50.6 | – | 51.9 | – | 55.8 | 61.0 | – | 64.9 | – | – | 100 |
|  | % | – | 5.2 | – | – | 7.8 | – | 27.3 | – | 9.1 | – | 1.3 | – | 1.3 | – | 3.9 | 5.2 | – | 3.9 | – | – | 35.1 |
| CEC | N | – | – | – | – | – | – | – | – | 1 | – | 7 | – | 29 | 15 | 2 | 2 | 21 | – | – | – | – |
|  | Cum. % | – | – | – | – | – | – | – | – | 1.3 | – | 10.4 | – | 48.1 | 67.5 | 70.1 | 72.7 | 100 | – | – | – | – |
|  | % | – | – | – | – | – | – | – | – | 1.3 | – | 9.1 | – | 37.7 | 19.5 | 2.6 | 2.6 | 27.3 | – | – | – | – |
| CDR | N | – | 1 | – | – | 6 | – | 26 | – | 14 | – | 7 | – | 1 | 2 | 1 | 4 | – | 10 | 5 | – | – |
|  | Cum. % | – | 1.3 | – | – | 9.1 | – | 42.9 | – | 61.0 | – | 70.1 | – | 71.4 | 74.0 | 75.3 | 80.5 | – | 93.5 | 100 | – | – |
|  | % | – | 1.3 | – | – | 7.8 | – | 33.8 | – | 18.2 | – | 9.1 | – | 1.3 | 2.6 | 1.3 | 5.2 | – | 13.0 | 6.5 | – | – |
| CFM | N | – | – | – | – | – | – | – | – | – | 37 | – | – | 11 | 1 | 5 | 1 | – | 6 | – | 3 | 13 |
|  | Cum. % | – | – | – | – | – | – | – | – | – | 48.1 | – | – | 62.3 | 63.6 | 70.1 | 71.4 | – | 79.2 | – | 83.1 | 100 |
|  | % | – | – | – | – | – | – | – | – | – | 48.1 | – | – | 14.3 | 1.3 | 6.5 | 1.3 | – | 7.8 | – | 3.9 | 16.9 |
| CTX | N | 3 | – | 20 | – | 15 | – | 10 | – | 5 | – | 1 | – | 7 | 7 | 5 | – | 4 | – | – | – | – |
|  | Cum. % | 3.9 | – | 29.9 | – | 49.4 | – | 62.3 | – | 68.8 | – | 70.1 | – | 79.2 | 88.3 | 94.8 | – | 100 | – | – | – | – |
|  | % | 3.9 | – | 26.0 | – | 19.5 | – | 13.0 | – | 6.5 | – | 1.3 | – | 9.1 | 9.1 | 6.5 | – | 5.2 | – | – | – | – |
| CPD | N | – | 5 | – | – | 27 | – | 11 | – | 8 | – | 3 | – | 4 | 1 | 6 | 4 | 8 | – | – | – | – |
|  | Cum. % | – | 6.5 | – | – | 41.6 | – | 55.8 | – | 66.2 | – | 70.1 | – | 75.3 | 76.6 | 84.4 | 89.6 | 100 | – | – | – | – |
|  | % | – | 6.5 | – | – | 35.1 | – | 14.3 | – | 10.4 | – | 3.9 | – | 5.2 | 1.3 | 7.8 | 5.2 | 10.4 | – | – | – | – |
| CTB | N | – | – | – | – | – | – | – | – | – | – | – | 2 | – | 1 | 2 | 32 | – | 13 | – | 2 | 25 |
|  | Cum. % | – | – | – | – | – | – | – | – | – | – | – | 2.6 | – | 3.9 | 6.5 | 48.1 | – | 64.9 | – | 67.5 | 100 |
|  | % | – | – | – | – | – | – | – | – | – | – | – | 2.6 | – | 1.3 | 2.6 | 41.6 | – | 16.9 | – | 2.6 | 32.5 |
| CRO | N | 2 | – | 12 | – | 20 | – | 14 | – | 4 | – | 2 | – | 5 | 10 | 5 | 2 | 1 | – | – | – | – |
|  | Cum. % | 2.6 | – | 18.2 | – | 44.2 | – | 62.3 | – | 67.5 | – | 70.1 | – | 76.6 | 89.6 | 96.1 | 98.7 | 100 | – | – | – | – |
|  | % | 2.6 | – | 15.6 | – | 26.0 | – | 18.2 | – | 5.2 | – | 2.6 | – | 6.5 | 13.0 | 6.5 | 2.6 | 1.3 | – | – | – | – |
| CXM | N | 3 | – | 13 | – | 14 | – | 7 | – | 10 | – | 5 | – | 4 | 2 | 2 | 7 | – | 7 | 3 | – | – |
|  | Cum. % | 3.9 | – | 20.8 | – | 39.0 | – | 48.1 | – | 61.0 | – | 67.5 | – | 72.7 | 75.3 | 77.9 | 87.0 | – | 96.1 | 100 | – | – |
|  | % | 3.9 | – | 16.9 | – | 18.2 | – | 9.1 | – | 13.0 | – | 6.5 | – | 5.2 | 2.6 | 2.6 | 9.1 | – | 9.1 | 3.9 | – | – |
| CLR | N | – | 20 | – | – | 19 | – | 1 | – | – | – | 1 | – | 2 | 2 | 4 | 5 | – | 2 | – | 1 | 20 |
|  | Cum. % | – | 26.0 | – | – | 50.6 | – | 51.9 | – | – | – | 53.2 | – | 55.8 | 58.4 | 63.6 | 70.1 | – | 72.7 | – | 74.0 | 100 |
|  | % | – | 26.0 | – | – | 24.7 | – | 1.3 | – | – | – | 1.3 | – | 2.6 | 2.6 | 5.2 | 6.5 | – | 2.6 | – | 1.3 | 26.0 |
| DOX | N | 1 | – | – | – | 7 | – | 34 | – | 6 | – | 2 | – | 1 | – | 3 | 14 | 9 | – | – | – | – |
|  | Cum. % | 1.3 | – | – | – | 10.4 | – | 54.5 | – | 62.3 | – | 64.9 | – | 66.2 | – | 70.1 | 88.3 | 100 | – | – | – | – |
|  | % | 1.3 | – | – | – | 9.1 | – | 44.2 | – | 7.8 | – | 2.6 | – | 1.3 | – | 3.9 | 18.2 | 11.7 | – | – | – | – |
| ERY | N | – | 10 | – | – | 21 | – | 8 | – | – | – | 1 | – | 1 | – | 4 | 3 | – | 5 | – | – | 24 |
|  | Cum. % | – | 13.0 | – | – | 40.3 | – | 50.6 | – | – | – | 51.9 | – | 53.2 | – | 58.4 | 62.3 | – | 68.8 | – | – | 100 |
|  | % | – | 13.0 | – | – | 27.3 | – | 10.4 | – | – | – | 1.3 | – | 1.3 | – | 5.2 | 3.9 | – | 6.5 | – | – | 31.2 |
| LVX | N | – | – | – | – | – | – | – | 1 | – | – | – | – | 22 | 52 | 2 | – | – | – | – | – | – |
|  | Cum. % | – | – | – | – | – | – | – | 1.3 | – | – | – | – | 29.9 | 97.4 | 100 | – | – | – | – | – | – |
|  | % | – | – | – | – | – | – | – | 1.3 | – | – | – | – | 28.6 | 67.5 | 2.6 | – | – | – | – | – | – |
| MXF | N | – | – | – | 5 | – | – | 29 | – | 43 | – | – | – | – | – | – | – | – | – | – | – | – |
|  | Cum. % | – | – | – | 6.5 | – | – | 44.2 | – | 100 | – | – | – | – | – | – | – | – | – | – | – | – |
|  | % | – | – | – | 6.5 | – | – | 37.7 | – | 55.8 | – | – | – | – | – | – | – | – | – | – | – | – |
| PEN | N | 4 | – | 24 | – | 16 | – | 6 | – | 3 | – | 2 | – | 2 | 3 | 11 | 6 | – | – | – | – | – |
|  | Cum. % | 5.2 | – | 36.4 | – | 57.1 | – | 64.9 | – | 68.8 | – | 71.4 | – | 74.0 | 77.9 | 92.2 | 100 | – | – | – | – | – |
|  | % | 5.2 | – | 31.2 | – | 20.8 | – | 7.8 | – | 3.9 | – | 2.6 | – | 2.6 | 3.9 | 14.3 | 7.8 | – | – | – | – | – |
| TET | N | – | – | – | 2 | – | – | 2 | – | 35 | – | 9 | – | 1 | 1 | – | 1 | 26 | – | – | – | – |
|  | Cum. % | – | – | – | 2.6 | – | – | 5.2 | – | 50.6 | – | 62.3 | – | 63.6 | 64.9 | – | 66.2 | 100 | – | – | – | – |
|  | % | – | – | – | 2.6 | – | – | 2.6 | – | 45.5 | – | 11.7 | – | 1.3 | 1.3 | – | 1.3 | 33.8 | – | – | – | – |
| SXT | N | – | – | – | – | – | 4 | – | – | 8 | – | 31 | – | 5 | 4 | 2 | 8 | – | 15 | – | – | – |
|  | Cum. % | – | – | – | – | – | 5.2 | – | – | 15.6 | – | 55.8 | – | 62.3 | 67.5 | 70.1 | 80.5 | – | 100 | – | – | – |
|  | % | – | – | – | – | – | 5.2 | – | – | 10.4 | – | 40.3 | – | 6.5 | 5.2 | 2.6 | 10.4 | – | 19.5 | – | – | – |

–, not applicable; AMC, amoxicillin/clavulanic acid; AMX, amoxicillin; AZM, azithromycin; CDR, cefdinir; CEC, cefaclor; CFM, cefixime; CLR, clarithromycin; CPD, cefpodoxime; CRO, ceftriaxone; CTB, ceftibuten; CTX, cefotaxime; Cum., cumulative; CXM, cefuroxime; DOX, doxycycline; ERY, erythromycin; LVX, levofloxacin; MXF, moxifloxacin; PEN, penicillin; SXT, trimethoprim/sulfamethoxazole; TET, tetracycline.

Bold vertical bars in table correspond to the CLSI-susceptible breakpoints.**Supplementary Table 2.** MIC distribution data for *S. pneumoniae* isolates (*n* = 176) from Spain

|  |  | Number of isolates at MIC (mg/L) | | | | | | | | | | | | | | | | | | | | |
| --- | --- | --- | --- | --- | --- | --- | --- | --- | --- | --- | --- | --- | --- | --- | --- | --- | --- | --- | --- | --- | --- | --- |
| Antimicrobial |  | ≤0.008 | ≤0.015 | 0.015 | ≤0.03 | 0.03 | ≤0.06 | 0.06 | ≤0.12 | 0.12 | ≤0.25 | 0.25 | ≤0.5 | 0.5 | 1 | 2 | 4 | >4 | 8 | >8 | 16 | >16 |
| AMX | N | 3 | – | 65 | – | 46 | – | 16 | – | 7 | – | 8 | – | 2 | 2 | 13 | 6 | – | 8 | – | – | – |
|  | Cum. % | 1.7 | – | 38.6 | – | 64.8 | – | 73.9 | – | 77.8 | – | 82.4 | – | 83.5 | 84.7 | 92.0 | 95.5 | – | 100 | – | – | – |
|  | % | 1.7 | – | 36.9 | – | 26.1 | – | 9.1 | – | 4.0 | – | 4.5 | – | 1.1 | 1.1 | 7.4 | 3.4 | – | 4.5 | – | – | – |
| AMC (2:1) | N | 3 | – | 59 | – | 53 | – | 14 | – | 8 | – | 8 | – | 2 | 2 | 13 | 6 | – | 8 | – | – | – |
|  | Cum. % | 1.7 | – | 35.2 | – | 65.3 | – | 73.3 | – | 77.8 | – | 82.4 | – | 83.5 | 84.7 | 92.0 | 95.5 | – | 100 | – | – | – |
|  | % | 1.7 | – | 33.5 | – | 30.1 | – | 8.0 | – | 4.5 | – | 4.5 | – | 1.1 | 1.1 | 7.4 | 3.4 | – | 4.5 | – | – | – |
| AMC [2 mg/L] | N | 3 | – | 5 | – | 56 | – | 49 | – | 15 | – | 9 | – | 6 | 4 | – | 2 | – | 13 | 14 | – | – |
|  | Cum. % | 1.7 | – | 4.5 | – | 36.4 | – | 64.2 | – | 72.7 | – | 77.8 | – | 81.3 | 83.5 | – | 84.7 | – | 92.0 | 100 | – | – |
|  | % | 1.7 | – | 2.8 | – | 31.8 | – | 27.8 | – | 8.5 | – | 5.1 | – | 3.4 | 2.3 | – | 1.1 | – | 7.4 | 8.0 | – | – |
| AZM | N | – | 14 | – | – | 62 | – | 58 | – | 1 | – | – | – | 1 | 2 | 2 | 1 | – | 4 | – | – | 31 |
|  | Cum. % | – | 8.0 | – | – | 43.2 | – | 76.1 | – | 76.7 | – | – | – | 77.3 | 78.4 | 79.5 | 80.1 | – | 82.4 | – | – | 100 |
|  | % | – | 8.0 | – | – | 35.2 | – | 33.0 | – | 0.6 | – | – | – | 0.6 | 1.1 | 1.1 | 0.6 | – | 2.3 | – | – | 17.6 |
| CEC | N | – | – | – | – | 1 | – | – | – | – | – | 12 | – | 92 | 27 | 9 | 4 | 31 | – | – | – | – |
|  | Cum. % | – | – | – | – | 0.6 | – | – | – | – | – | 7.4 | – | 59.7 | 75.0 | 80.1 | 82.4 | 100 | – | – | – | – |
|  | % | – | – | – | – | 0.6 | – | – | – | – | – | 6.8 | – | 52.3 | 15.3 | 5.1 | 2.3 | 17.6 | – | – | – | – |
| CDR | N | – | – | – | – | 9 | – | 98 | – | 20 | – | 12 | – | 7 | – | 5 | 12 | – | 9 | 4 | – | – |
|  | Cum. % | – | – | – | – | 5.1 | – | 60.8 | – | 72.2 | – | 79.0 | – | 83.0 | – | 85.8 | 92.6 | – | 97.7 | 100 | – | – |
|  | % | – | – | – | – | 5.1 | – | 55.7 | – | 11.4 | – | 6.8 | – | 4.0 | – | 2.8 | 6.8 | – | 5.1 | 2.3 | – | – |
| CFM | N | – | – | – | – | – | – | – | – | – | 102 | – | – | 8 | 20 | 9 | 7 | – | 7 | – | 14 | 9 |
|  | Cum. % | – | – | – | – | – | – | – | – | – | 58.0 | – | – | 62.5 | 73.9 | 79.0 | 83.0 | – | 86.9 | – | 94.9 | 100 |
|  | % | – | – | – | – | – | – | – | – | – | 58.0 | – | – | 4.5 | 11.4 | 5.1 | 4.0 | – | 4.0 | – | 8.0 | 5.1 |
| CTX | N | 7 | – | 59 | – | 40 | – | 22 | – | 13 | – | 5 | – | 10 | 12 | 8 | – | – | – | – | – | – |
|  | Cum. % | 4.0 | – | 37.5 | – | 60.2 | – | 72.7 | – | 80.1 | – | 83.0 | – | 88.6 | 95.5 | 100 | – | – | – | – | – | – |
|  | % | 4.0 | – | 33.5 | – | 22.7 | – | 12.5 | – | 7.4 | – | 2.8 | – | 5.7 | 6.8 | 4.5 | – | – | – | – | – | – |
| CPD | N | – | 14 | – | – | 88 | – | 15 | – | 21 | – | 7 | – | 1 | 5 | 16 | 8 | 1 | – | – | – | – |
|  | Cum. % | – | 8.0 | – | – | 58.0 | – | 66.5 | – | 78.4 | – | 82.4 | – | 83.0 | 85.8 | 94.9 | 99.4 | 100 | – | – | – | – |
|  | % | – | 8.0 | – | – | 50.0 | – | 8.5 | – | 11.9 | – | 4.0 | – | 0.6 | 2.8 | 9.1 | 4.5 | 0.6 | – | – | – | – |
| CTB | N | – | – | – | – | – | – | – | – | – | – | – | – | – | 2 | 14 | 85 | – | 11 | – | 18 | 46 |
|  | Cum. % | – | – | – | – | – | – | – | – | – | – | – | – | – | 1.1 | 9.1 | 57.4 | – | 63.6 | – | 73.9 | 100 |
|  | % | – | – | – | – | – | – | – | – | – | – | – | – | – | 1.1 | 8.0 | 48.3 | – | 6.3 | – | 10.2 | 26.1 |
| CRO | N | 2 | – | 56 | – | 46 | – | 23 | – | 14 | – | 5 | – | 10 | 13 | 7 | – | – | – | – | – | – |
|  | Cum. % | 1.1 | – | 33.0 | – | 59.1 | – | 72.2 | – | 80.1 | – | 83.0 | – | 88.6 | 96.0 | 100 | – | – | – | – | – | – |
|  | % | 1.1 | – | 31.8 | – | 26.1 | – | 13.1 | – | 8.0 | – | 2.8 | – | 5.7 | 7.4 | 4.0 | – | – | – | – | – | – |
| CXM | N | 2 | – | 39 | – | 61 | – | 2 | – | 17 | – | 17 | – | 7 | 2 | 9 | 13 | – | 7 | – | – | – |
|  | Cum. % | 1.1 | – | 23.3 | – | 58.0 | – | 59.1 | – | 68.8 | – | 78.4 | – | 82.4 | 83.5 | 88.6 | 96.0 | – | 100 | – | – | – |
|  | % | 1.1 | – | 22.2 | – | 34.7 | – | 1.1 | – | 9.7 | – | 9.7 | – | 4.0 | 1.1 | 5.1 | 7.4 | – | 4.0 | – | – | – |
| CLR | N | – | 108 | – | – | 27 | – | – | – | – | – | 1 | – | 2 | 2 | 2 | 3 | – | 2 | – | – | 29 |
|  | Cum. % | – | 61.4 | – | – | 76.7 | – | – | – | – | – | 77.3 | – | 78.4 | 79.5 | 80.7 | 82.4 | – | 83.5 | – | – | 100 |
|  | % | – | 61.4 | – | – | 15.3 | – | – | – | – | – | 0.6 | – | 1.1 | 1.1 | 1.1 | 1.7 | – | 1.1 | – | – | 16.5 |
| DOX | N | – | – | 1 | – | 25 | – | 100 | – | 14 | – | 2 | – | 2 | 2 | 3 | 13 | 14 | – | – | – | – |
|  | Cum. % | – | – | 0.6 | – | 14.8 | – | 71.6 | – | 79.5 | – | 80.7 | – | 81.8 | 83.0 | 84.7 | 92.0 | 100 | – | – | – | – |
|  | % | – | – | 0.6 | – | 14.2 | – | 56.8 | – | 8.0 | – | 1.1 | – | 1.1 | 1.1 | 1.7 | 7.4 | 8.0 | – | – | – | – |
| ERY | N | – | 49 | – | – | 85 | – | 1 | – | – | – | – | – | – | 3 | 2 | 2 | – | 2 | – | 2 | 30 |
|  | Cum. % | – | 27.8 | – | – | 76.1 | – | 76.7 | – | – | – | – | – | – | 78.4 | 79.5 | 80.7 | – | 81.8 | – | 83.0 | 100 |
|  | % | – | 27.8 | – | – | 48.3 | – | 0.6 | – | – | – | – | – | – | 1.7 | 1.1 | 1.1 | – | 1.1 | – | 1.1 | 17.0 |
| LVX | N | – | – | – | – | – | – | – | – | – | – | 2 | – | 45 | 124 | 4 | – | – | – | 1 | – | – |
|  | Cum. % | – | – | – | – | – | – | – | – | – | – | 1.1 | – | 26.7 | 97.2 | 99.4 | – | – | – | 100 | – | – |
|  | % | – | – | – | – | – | – | – | – | – | – | 1.1 | – | 25.6 | 70.5 | 2.3 | – | – | – | 0.6 | – | – |
| MXF | N | – | – | – | 11 | – | – | 68 | – | 94 | – | 2 | – | – | – | – | 1 | – | – | – | – | – |
|  | Cum. % | – | – | – | 6.3 | – | – | 44.9 | – | 98.3 | – | 99.4 | – | – | – | – | 100 | – | – | – | – | – |
|  | % | – | – | – | 6.3 | – | – | 38.6 | – | 53.4 | – | 1.1 | – | – | – | – | 0.6 | – | – | – | – | – |
| PEN | N | 9 | – | 82 | – | 15 | – | 9 | – | 8 | – | 18 | – | 5 | 10 | 12 | 8 | – | – | – | – | – |
|  | Cum. % | 5.1 | – | 51.7 | – | 60.2 | – | 65.3 | – | 69.9 | – | 80.1 | – | 83.0 | 88.6 | 95.5 | 100 | – | – | – | – | – |
|  | % | 5.1 | – | 46.6 | – | 8.5 | – | 5.1 | – | 4.5 | – | 10.2 | – | 2.8 | 5.7 | 6.8 | 4.5 | – | – | – | – | – |
| TET | N | – | – | – | – | – | – | 6 | – | 114 | – | 19 | – | 4 | – | 2 | 1 | 30 | – | – | – | – |
|  | Cum. % | – | – | – | – | – | – | 3.4 | – | 68.2 | – | 79.0 | – | 81.3 | – | 82.4 | 83.0 | 100 | – | – | – | – |
|  | % | – | – | – | – | – | – | 3.4 | – | 64.8 | – | 10.8 | – | 2.3 | – | 1.1 | 0.6 | 17.0 | – | – | – | – |
| SXT | N | – | – | – | – | – | 5 | – | – | 33 | – | 70 | – | 10 | 11 | 6 | 23 | – | 14 | 4 | – | – |
|  | Cum. % | – | – | – | – | – | 2.8 | – | – | 21.6 | – | 61.4 | – | 67.0 | 73.3 | 76.7 | 89.8 | – | 97.7 | 100 | – | – |
|  | % | – | – | – | – | – | 2.8 | – | – | 18.8 | – | 39.8 | – | 5.7 | 6.3 | 3.4 | 13.1 | – | 8.0 | 2.3 | – | – |

–, not applicable; AMC, amoxicillin/clavulanic acid; AMX, amoxicillin; AZM, azithromycin; CDR, cefdinir; CEC, cefaclor; CFM, cefixime; CLR, clarithromycin; CPD, cefpodoxime; CRO, ceftriaxone; CTB, ceftibuten; CTX, cefotaxime; Cum., cumulative; CXM, cefuroxime; DOX, doxycycline; ERY, erythromycin; LVX, levofloxacin; MXF, moxifloxacin; PEN, penicillin; SXT, trimethoprim/sulfamethoxazole; TET, tetracycline.

Bold vertical bars in table correspond to the CLSI-susceptible breakpoints.

**Supplementary Table 3.** MIC distribution data for *H. influenzae* isolates (*n* = 249) from Italy

|  |  | Number of isolates at MIC (mg/L) | | | | | | | | | | | | | | | | | | | | | | | | | | | | | |
| --- | --- | --- | --- | --- | --- | --- | --- | --- | --- | --- | --- | --- | --- | --- | --- | --- | --- | --- | --- | --- | --- | --- | --- | --- | --- | --- | --- | --- | --- | --- | --- |
| Antimicrobial |  | ≤0.001 | ≤0.002 | 0.002 | ≤0.004 | 0.004 | ≤0.008 | 0.008 | ≤0.015 | 0.015 | ≤0.03 | 0.03 | ≤0.06 | 0.06 | ≤0.12 | 0.12 | ≤0.25 | 0.25 | 0.5 | 1 | 2 | 4 | >4 | 8 | >8 | 16 | 32 | >32 | 64 | 128 | >128 |
| AMX | N | – | – | – | – | – | – | – | – | – | 2 | – | – | 2 | – | 7 | – | 66 | 81 | 23 | 23 | 9 | – | 5 | – | 9 | 8 | – | 6 | 8 | – |
|  | Cum. % | – | – | – | – | – | – | – | – | – | 0.8 | – | – | 1.6 | – | 4.4 | – | 30.9 | 63.5 | 72.7 | 81.9 | 85.5 | – | 87.6 | – | 91.2 | 94.4 | – | 96.8 | 100 | – |
|  | % | – | – | – | – | – | – | – | – | – | 0.8 | – | – | 0.8 | – | 2.8 | – | 26.5 | 32.5 | 9.2 | 9.2 | 3.6 | – | 2.0 | – | 3.6 | 3.2 | – | 2.4 | 3.2 | – |
| AMC (2:1) | N | – | – | – | – | – | – | – | – | – | 2 | – | – | 1 | – | 8 | – | 61 | 91 | 41 | 28 | 13 | – | 4 | – | – | – | – | – | – | – |
|  | Cum. % | – | – | – | – | – | – | – | – | – | 0.8 | – | – | 1.2 | – | 4.4 | – | 28.9 | 65.5 | 81.9 | 93.2 | 98.4 | – | 100 | – | – | – | – | – | – | – |
|  | % | – | – | – | – | – | – | – | – | – | 0.8 | – | – | 0.4 | – | 3.2 | – | 24.5 | 36.5 | 16.5 | 11.2 | 5.2 | – | 1.6 | – | – | – | – | – | – | – |
| AMC [2mg/L] | N | – | – | – | – | – | – | – | – | – | 5 | – | – | 1 | – | 17 | – | 88 | 74 | 30 | 27 | 4 | – | 3 | – | – | – | – | – | – | – |
|  | Cum. % | – | – | – | – | – | – | – | – | – | 2.0 | – | – | 2.4 | – | 9.2 | – | 44.6 | 74.3 | 86.3 | 97.2 | 98.8 | – | 100 | – | – | – | – | – | – | – |
|  | % | – | – | – | – | – | – | – | – | – | 2.0 | – | – | 0.4 | – | 6.8 | – | 35.3 | 29.7 | 12.0 | 10.8 | 1.6 | – | 1.2 | – | – | – | – | – | – | – |
| AMP | N | – | – | – | – | – | – | – | – | – | 4 | – | – | 4 | – | 64 | – | 75 | 22 | 37 | 9 | 1 | – | 3 | – | 10 | 6 | – | 6 | 7 | 1 |
|  | Cum. % | – | – | – | – | – | – | – | – | – | 1.6 | – | – | 3.2 | – | 28.9 | – | 59.0 | 67.9 | 82.7 | 86.3 | 86.7 | – | 88.0 | – | 92.0 | 94.4 | – | 96.8 | 99.6 | 100 |
|  | % | – | – | – | – | – | – | – | – | – | 1.6 | – | – | 1.6 | – | 25.7 | – | 30.1 | 8.8 | 14.9 | 3.6 | 0.4 | – | 1.2 | – | 4.0 | 2.4 | – | 2.4 | 2.8 | 0.4 |
| AZM | N | – | – | – | – | – | – | – | – | – | – | – | – | – | 6 | – | – | 12 | 104 | 91 | 31 | 2 | – | – | 3 | – | – | – | – | – | – |
|  | Cum. % | – | – | – | – | – | – | – | – | – | – | – | – | – | 2.4 | – | – | 7.2 | 49.0 | 85.5 | 98.0 | 98.8 | – | – | 100 | – | – | – | – | – | – |
|  | % | – | – | – | – | – | – | – | – | – | – | – | – | – | 2.4 | – | – | 4.8 | 41.8 | 36.5 | 12.4 | 0.8 | – | – | 1.2 | – | – | – | – | – | – |
| CEC | N | – | – | – | – | – | – | – | – | – | – | – | – | – | – | – | 3 | – | 13 | 73 | 83 | 36 | – | 33 | – | 2 | 6 | – | – | – | – |
|  | Cum. % | – | – | – | – | – | – | – | – | – | – | – | – | – | – | – | 1.2 | – | 6.4 | 35.7 | 69.1 | 83.5 | – | 96.8 | – | 97.6 | 100 | – | – | – | – |
|  | % | – | – | – | – | – | – | – | – | – | – | – | – | – | – | – | 1.2 | – | 5.2 | 29.3 | 33.3 | 14.5 | – | 13.3 | – | 0.8 | 2.4 | – | – | – | – |
| CDR | N | – | – | – | – | – | – | – | – | – | – | – | 2 | – | – | 47 | – | 129 | 45 | 22 | 4 | – | – | – | – | – | – | – | – | – | – |
|  | Cum. % | – | – | – | – | – | – | – | – | – | – | – | 0.8 | – | – | 19.7 | – | 71.5 | 89.6 | 98.4 | 100 | – | – | – | – | – | – | – | – | – | – |
|  | % | – | – | – | – | – | – | – | – | – | – | – | 0.8 | – | – | 18.9 | – | 51.8 | 18.1 | 8.8 | 1.6 | – | – | – | – | – | – | – | – | – | – |
| CFM | N | – | – | – | – | – | 3 | – | – | 69 | – | 148 | – | 19 | – | 2 | – | 6 | – | 2 | – | – | – | – | – | – | – | – | – | – | – |
|  | Cum. % | – | – | – | – | – | 1.2 | – | – | 28.9 | – | 88.4 | – | 96.0 | – | 96.8 | – | 99.2 | – | 100 | – | – | – | – | – | – | – | – | – | – | – |
|  | % | – | – | – | – | – | 1.2 | – | – | 27.7 | – | 59.4 | – | 7.6 | – | 0.8 | – | 2.4 | – | 0.8 | – | – | – | – | – | – | – | – | – | – | – |
| CTX | N | – | 13 | – | – | 25 | – | 76 | – | 72 | – | 43 | – | 14 | – | 5 | – | – | 1 | – | – | – | – | – | – | – | – | – | – | – | – |
|  | Cum. % | – | 5.2 | – | – | 15.3 | – | 45.8 | – | 74.7 | – | 92.0 | – | 97.6 | – | 99.6 | – | – | 100 | – | – | – | – | – | – | – | – | – | – | – | – |
|  | % | – | 5.2 | – | – | 10.0 | – | 30.5 | – | 28.9 | – | 17.3 | – | 5.6 | – | 2.0 | – | – | 0.4 | – | – | – | – | – | – | – | – | – | – | – | – |
| CPD | N | – | – | – | – | – | – | – | 10 | – | – | 74 | – | 94 | – | 38 | – | 25 | 1 | 2 | 5 | – | – | – | – | – | – | – | – | – | – |
|  | Cum. % | – | – | – | – | – | – | – | 4.0 | – | – | 33.7 | – | 71.5 | – | 86.7 | – | 96.8 | 97.2 | 98.0 | 100 | – | – | – | – | – | – | – | – | – | – |
|  | % | – | – | – | – | – | – | – | 4.0 | – | – | 29.7 | – | 37.8 | – | 15.3 | – | 10.0 | 0.4 | 0.8 | 2.0 | – | – | – | – | – | – | – | – | – | – |
| CTB | N | – | – | – | – | – | 1 | – | – | 2 | – | 52 | – | 117 | – | 34 | – | 29 | 5 | 3 | 4 | 1 | 1 | – | – | – | – | – | – | – | – |
|  | Cum. % | – | – | – | – | – | 0.4 | – | – | 1.2 | – | 22.1 | – | 69.1 | – | 82.7 | – | 94.4 | 96.4 | 97.6 | 99.2 | 99.6 | 100 | – | – | – | – | – | – | – | – |
|  | % | – | – | – | – | – | 0.4 | – | – | 0.8 | – | 20.9 | – | 47.0 | – | 13.7 | – | 11.6 | 2.0 | 1.2 | 1.6 | 0.4 | 0.4 | – | – | – | – | – | – | – | – |
| CRO | N | 3 | – | 62 | – | 112 | – | 38 | – | 23 | – | 6 | – | – | – | 4 | – | 1 | – | – | – | – | – | – | – | – | – | – | – | – | – |
|  | Cum. % | 1.2 | – | 26.1 | – | 71.1 | – | 86.3 | – | 95.6 | – | 98.0 | – | – | – | 99.6 | – | 100 | – | – | – | – | – | – | – | – | – | – | – | – | – |
|  | % | 1.2 | – | 24.9 | – | 45.0 | – | 15.3 | – | 9.2 | – | 2.4 | – | – | – | 1.6 | – | 0.4 | – | – | – | – | – | – | – | – | – | – | – | – | – |
| CXM | N | – | – | – | – | – | – | – | – | – | 4 | – | – | 2 | – | 2 | – | 35 | 114 | 48 | 40 | 4 | – | – | – | – | – | – | – | – | – |
|  | Cum. % | – | – | – | – | – | – | – | – | – | 1.6 | – | – | 2.4 | – | 3.2 | – | 17.3 | 63.1 | 82.3 | 98.4 | 100 | – | – | – | – | – | – | – | – | – |
|  | % | – | – | – | – | – | – | – | – | – | 1.6 | – | – | 0.8 | – | 0.8 | – | 14.1 | 45.8 | 19.3 | 16.1 | 1.6 | – | – | – | – | – | – | – | – | – |
| CLR | N | – | – | – | – | – | – | – | – | – | – | – | – | – | – | – | 1 | – | 3 | 1 | 12 | 138 | – | 88 | – | 2 | 1 | 3 | – | – | – |
|  | Cum. % | – | – | – | – | – | – | – | – | – | – | – | – | – | – | – | 0.4 | – | 1.6 | 2.0 | 6.8 | 62.2 | – | 97.6 | – | 98.4 | 98.8 | 100 | – | – | – |
|  | % | – | – | – | – | – | – | – | – | – | – | – | – | – | – | – | 0.4 | – | 1.2 | 0.4 | 4.8 | 55.4 | – | 35.3 | – | 0.8 | 0.4 | 1.2 | – | – | – |
| LVX | N | – | – | – | 3 | – | – | 10 | – | 201 | – | 24 | – | 1 | – | 1 | – | – | 6 | – | – | – | – | 1 | 2 | – | – | – | – | – | – |
|  | Cum. % | – | – | – | 1.2 | – | – | 5.2 | – | 85.9 | – | 95.6 | – | 96.0 | – | 96.4 | – | – | 98.8 | – | – | – | – | 99.2 | 100 | – | – | – | – | – | – |
|  | % | – | – | – | 1.2 | – | – | 4.0 | – | 80.7 | – | 9.6 | – | 0.4 | – | 0.4 | – | – | 2.4 | – | – | – | – | 0.4 | 0.8 | – | – | – | – | – | – |
| MXF | N | – | – | – | 4 | – | – | 16 | – | 158 | – | 52 | – | 8 | – | 2 | – | 1 | 3 | 2 | – | – | – | – | 3 | – | – | – | – | – | – |
|  | Cum. % | – | – | – | 1.6 | – | – | 8.0 | – | 71.5 | – | 92.4 | – | 95.6 | – | 96.4 | – | 96.8 | 98.0 | 98.8 | – | – | – | – | 100 | – | – | – | – | – | – |
|  | % | – | – | – | 1.6 | – | – | 6.4 | – | 63.5 | – | 20.9 | – | 3.2 | – | 0.8 | – | 0.4 | 1.2 | 0.8 | – | – | – | – | 1.2 | – | – | – | – | – | – |
| TET | N | – | – | – | – | – | – | – | – | – | – | – | – | – | 7 | – | – | 194 | 46 | – | 1 | – | – | 1 | – | – | – | – | – | – | – |
|  | Cum. % | – | – | – | – | – | – | – | – | – | – | – | – | – | 2.8 | – | – | 80.7 | 99.2 | – | 99.6 | – | – | 100 | – | – | – | – | – | – | – |
|  | % | – | – | – | – | – | – | – | – | – | – | – | – | – | 2.8 | – | – | 77.9 | 18.5 | – | 0.4 | – | – | 0.4 | – | – | – | – | – | – | – |
| SXT | N | – | – | – | – | – | 3 | – | – | 5 | – | 20 | – | 75 | – | 53 | – | 18 | 3 | 4 | 7 | 21 | – | 19 | 21 | – | – | – | – | – | – |
|  | Cum. % | – | – | – | – | – | 1.2 | – | – | 3.2 | – | 11.2 | – | 41.4 | – | 62.7 | – | 69.9 | 71.1 | 72.7 | 75.5 | 83.9 | – | 91.6 | 100 | – | – | – | – | – | – |
|  | % | – | – | – | – | – | 1.2 | – | – | 2.0 | – | 8.0 | – | 30.1 | – | 21.3 | – | 7.2 | 1.2 | 1.6 | 2.8 | 8.4 | – | 7.6 | 8.4 | – | – | – | – | – | – |

–, not applicable; AMC, amoxicillin/clavulanic acid; AMX, amoxicillin; Antim., antimicrobial; AZM, azithromycin; CDN, cefdinir; CEC, cefaclor; CFM, cefixime; CLR, clarithromycin; CPD, cefpodoxime; CRO, ceftriaxone; CTB, ceftibuten; CTX, cefotaxime; Cum., cumulative; CXM, cefuroxime; DOX, doxycycline; ERY, erythromycin; LVX, levofloxacin; MXF, moxifloxacin; PEN, penicillin; SXT, trimethoprim/sulfamethoxazole; TET, tetracycline.

Bold vertical bars in table correspond to the CLSI-susceptible breakpoints.

**Supplementary Table 4.** MIC distribution data for *H. influenzae* isolates (*n* = 275) from Spain

|  |  | Number of isolates at MIC (mg/L) | | | | | | | | | | | | | | | | | | | | | | | | | | | | | |
| --- | --- | --- | --- | --- | --- | --- | --- | --- | --- | --- | --- | --- | --- | --- | --- | --- | --- | --- | --- | --- | --- | --- | --- | --- | --- | --- | --- | --- | --- | --- | --- |
| Antimicrobial |  | ≤0.001 | ≤0.002 | 0.002 | ≤0.004 | 0.004 | ≤0.008 | 0.008 | ≤0.015 | 0.015 | ≤0.03 | 0.03 | ≤0.06 | 0.06 | ≤0.12 | 0.12 | ≤0.25 | 0.25 | 0.5 | 1 | 2 | 4 | >4 | 8 | >8 | 16 | 32 | >32 | 64 | 128 | >128 |
| AMX | N | – | – | – | – | – | – | – | – | – | 2 | – | – | – | – | 4 | – | 56 | 108 | 22 | 30 | 9 | – | 6 | – | 6 | 12 | – | 12 | 4 | 4 |
|  | Cum. % | – | – | – | – | – | – | – | – | – | 0.7 | – | – | – | – | 2.2 | – | 22.5 | 61.8 | 69.8 | 80.7 | 84.0 | – | 86.2 | – | 88.4 | 92.7 | – | 97.1 | 98.5 | 100 |
|  | % | – | – | – | – | – | – | – | – | – | 0.7 | – | – | – | – | 1.5 | – | 20.4 | 39.3 | 8.0 | 10.9 | 3.3 | – | 2.2 | – | 2.2 | 4.4 | – | 4.4 | 1.5 | 1.5 |
| AMC (2:1) | N | – | – | – | – | – | – | – | – | – | 2 | – | – | – | – | – | – | 50 | 127 | 56 | 31 | 7 | – | 1 | – | 1 | – | – | – | – | – |
|  | Cum. % | – | – | – | – | – | – | – | – | – | 0.7 | – | – | – | – | – | – | 18.9 | 65.1 | 85.5 | 96.7 | 99.3 | – | 99.6 | – | 100 | – | – | – | – | – |
|  | % | – | – | – | – | – | – | – | – | – | 0.7 | – | – | – | – | – | – | 18.2 | 46.2 | 20.4 | 11.3 | 2.5 | – | 0.4 | – | 0.4 | – | – | – | – | – |
| AMC [2mg/L] | N | – | – | – | – | – | – | – | – | – | 2 | – | – | 2 | – | 11 | – | 103 | 98 | 33 | 22 | 3 | – | – | – | 1 | – | – | – | – | – |
|  | Cum. % | – | – | – | – | – | – | – | – | – | 0.7 | – | – | 1.5 | – | 5.5 | – | 42.9 | 78.5 | 90.5 | 98.5 | 99.6 | – | – | – | 100 | – | – | – | – | – |
|  | % | – | – | – | – | – | – | – | – | – | 0.7 | – | – | 0.7 | – | 4.0 | – | 37.5 | 35.6 | 12.0 | 8.0 | 1.1 | – | – | – | 0.4 | – | – | – | – | – |
| AMP | N | – | – | – | – | – | – | – | – | – | 2 | – | – | 2 | – | 59 | – | 103 | 20 | 37 | 3 | 4 | – | 6 | – | 6 | 10 | – | 4 | 15 | 4 |
|  | Cum. % | – | – | – | – | – | – | – | – | – | 0.7 | – | – | 1.5 | – | 22.9 | – | 60.4 | 67.6 | 81.1 | 82.2 | 83.6 | – | 85.8 | – | 88.0 | 91.6 | – | 93.1 | 98.5 | 100 |
|  | % | – | – | – | – | – | – | – | – | – | 0.7 | – | – | 0.7 | – | 21.5 | – | 37.5 | 7.3 | 13.5 | 1.1 | 1.5 | – | 2.2 | – | 2.2 | 3.6 | – | 1.5 | 5.5 | 1.5 |
| AZM | N | – | – | – | – | – | – | – | – | – | – | – | – | – | 9 | – | – | 17 | 89 | 125 | 28 | 2 | – | – | 5 | – | – | – | – | – | – |
|  | Cum. % | – | – | – | – | – | – | – | – | – | – | – | – | – | 3.3 | – | – | 9.5 | 41.8 | 87.3 | 97.5 | 98.2 | – | – | 100 | – | – | – | – | – | – |
|  | % | – | – | – | – | – | – | – | – | – | – | – | – | – | 3.3 | – | – | 6.2 | 32.4 | 45.5 | 10.2 | 0.7 | – | – | 1.8 | – | – | – | – | – | – |
| CEC | N | – | – | – | – | – | – | – | – | – | – | – | – | – | – | – | 3 | – | 14 | 73 | 103 | 56 | – | 19 | – | 6 | – | 1 | – | – | – |
|  | Cum. % | – | – | – | – | – | – | – | – | – | – | – | – | – | – | – | 1.1 | – | 6.2 | 32.7 | 70.2 | 90.5 | – | 97.5 | – | 99.6 | – | 100 | – | – | – |
|  | % | – | – | – | – | – | – | – | – | – | – | – | – | – | – | – | 1.1 | – | 5.1 | 26.5 | 37.5 | 20.4 | – | 6.9 | – | 2.2 | – | 0.4 | – | – | – |
| CDR | N | – | – | – | – | – | – | – | – | – | – | – | 11 | – | – | 40 | – | 157 | 42 | 23 | 2 | – | – | – | – | – | – | – | – | – | – |
|  | Cum. % | – | – | – | – | – | – | – | – | – | – | – | 4.0 | – | – | 18.5 | – | 75.6 | 90.9 | 99.3 | 100 | – | – | – | – | – | – | – | – | – | – |
|  | % | – | – | – | – | – | – | – | – | – | – | – | 4.0 | – | – | 14.5 | – | 57.1 | 15.3 | 8.4 | 0.7 | – | – | – | – | – | – | – | – | – | – |
| CFM | N | – | – | – | – | – | 12 | – | – | 58 | – | 162 | – | 30 | – | 4 | – | 5 | 3 | 1 | – | – | – | – | – | – | – | – | – | – | – |
|  | Cum. % | – | – | – | – | – | 4.4 | – | – | 25.5 | – | 84.4 | – | 95.3 | – | 96.7 | – | 98.5 | 99.6 | 100 | – | – | – | – | – | – | – | – | – | – | – |
|  | % | – | – | – | – | – | 4.4 | – | – | 21.1 | – | 58.9 | – | 10.9 | – | 1.5 | – | 1.8 | 1.1 | 0.4 | – | – | – | – | – | – | – | – | – | – | – |
| CTX | N | – | 28 | – | – | 12 | – | 85 | – | 79 | – | 49 | – | 19 | – | 2 | – | 1 | – | – | – | – | – | – | – | – | – | – | – | – | – |
|  | Cum. % | – | 10.2 | – | – | 14.5 | – | 45.5 | – | 74.2 | – | 92.0 | – | 98.9 | – | 99.6 | – | 100 | – | – | – | – | – | – | – | – | – | – | – | – | – |
|  | % | – | 10.2 | – | – | 4.4 | – | 30.9 | – | 28.7 | – | 17.8 | – | 6.9 | – | 0.7 | – | 0.4 | – | – | – | – | – | – | – | – | – | – | – | – | – |
| CPD | N | – | – | – | – | – | – | – | 12 | – | – | 85 | – | 112 | – | 29 | – | 31 | 6 | – | – | – | – | – | – | – | – | – | – | – | – |
|  | Cum. % | – | – | – | – | – | – | – | 4.4 | – | – | 35.3 | – | 76.0 | – | 86.5 | – | 97.8 | 100 | – | – | – | – | – | – | – | – | – | – | – | – |
|  | % | – | – | – | – | – | – | – | 4.4 | – | – | 30.9 | – | 40.7 | – | 10.5 | – | 11.3 | 2.2 | – | – | – | – | – | – | – | – | – | – | – | – |
| CTB | N | – | – | – | – | – | 5 | – | – | 3 | – | 47 | – | 134 | – | 36 | – | 36 | 5 | 4 | 4 | – | 1 | – | – | – | – | – | – | – | – |
|  | Cum. % | – | – | – | – | – | 1.8 | – | – | 2.9 | – | 20.0 | – | 68.7 | – | 81.8 | – | 94.9 | 96.7 | 98.2 | 99.6 | – | 100 | – | – | – | – | – | – | – | – |
|  | % | – | – | – | – | – | 1.8 | – | – | 1.1 | – | 17.1 | – | 48.7 | – | 13.1 | – | 13.1 | 1.8 | 1.5 | 1.5 | – | 0.4 | – | – | – | – | – | – | – | – |
| CRO | N | 5 | – | 66 | – | 129 | – | 40 | – | 24 | – | 9 | – | 2 | – | – | – | – | – | – | – | – | – | – | – | – | – | – | – | – | – |
|  | Cum. % | 1.8 | – | 25.8 | – | 72.7 | – | 87.3 | – | 96.0 | – | 99.3 | – | 100 | – | – | – | – | – | – | – | – | – | – | – | – | – | – | – | – | – |
|  | % | 1.8 | – | 24.0 | – | 46.9 | – | 14.5 | – | 8.7 | – | 3.3 | – | 0.7 | – | – | – | – | – | – | – | – | – | – | – | – | – | – | – | – | – |
| CXM | N | – | – | – | – | – | – | – | – | – | 7 | – | – | 4 | – | 6 | – | 33 | 139 | 51 | 34 | 1 | – | – | – | – | – | – | – | – | – |
|  | Cum. % | – | – | – | – | – | – | – | – | – | 2.5 | – | – | 4.0 | – | 6.2 | – | 18.2 | 68.7 | 87.3 | 99.6 | 100 | – | – | – | – | – | – | – | – | – |
|  | % | – | – | – | – | – | – | – | – | – | 2.5 | – | – | 1.5 | – | 2.2 | – | 12.0 | 50.5 | 18.5 | 12.4 | 0.4 | – | – | – | – | – | – | – | – | – |
| CLR | N | – | – | – | – | – | – | – | – | – | – | – | – | – | – | – | 4 | – | 5 | 5 | 30 | 111 | – | 110 | – | 4 | 1 | 5 | – | – | – |
|  | Cum. % | – | – | – | – | – | – | – | – | – | – | – | – | – | – | – | 1.5 | – | 3.3 | 5.1 | 16.0 | 56.4 | – | 96.4 | – | 97.8 | 98.2 | 100 | – | – | – |
|  | % | – | – | – | – | – | – | – | – | – | – | – | – | – | – | – | 1.5 | – | 1.8 | 1.8 | 10.9 | 40.4 | – | 40.0 | – | 1.5 | 0.4 | 1.8 | – | – | – |
| LVX | N | – | – | – | 7 | – | – | 18 | – | 221 | – | 20 | – | – | – | – | – | – | 2 | 1 | – | – | – | 1 | 5 | – | – | – | – | – | – |
|  | Cum. % | – | – | – | 2.5 | – | – | 9.1 | – | 89.5 | – | 96.7 | – | – | – | – | – | – | 97.5 | 97.8 | – | – | – | 98.2 | 100 | – | – | – | – | – | – |
|  | % | – | – | – | 2.5 | – | – | 6.5 | – | 80.4 | – | 7.3 | – | – | – | – | – | – | 0.7 | 0.4 | – | – | – | 0.4 | 1.8 | – | – | – | – | – | – |
| MXF | N | – | – | – | 8 | – | – | 25 | – | 161 | – | 69 | – | 3 | – | – | – | 1 | 2 | – | – | – | – | 1 | 5 | – | – | – | – | – | – |
|  | Cum. % | – | – | – | 2.9 | – | – | 12.0 | – | 70.5 | – | 95.6 | – | 96.7 | – | – | – | 97.1 | 97.8 | – | – | – | – | 98.2 | 100 | – | – | – | – | – | – |
|  | % | – | – | – | 2.9 | – | – | 9.1 | – | 58.5 | – | 25.1 | – | 1.1 | – | – | – | 0.4 | 0.7 | – | – | – | – | 0.4 | 1.8 | – | – | – | – | – | – |
| TET | N | – | – | – | – | – | – | – | – | – | – | – | – | – | 8 | – | – | 197 | 70 | – | – | – | – | – | – | – | – | – | – | – | – |
|  | Cum. % | – | – | – | – | – | – | – | – | – | – | – | – | – | 2.9 | – | – | 74.5 | 100 | – | – | – | – | – | – | – | – | – | – | – | – |
|  | % | – | – | – | – | – | – | – | – | – | – | – | – | – | 2.9 | – | – | 71.6 | 25.5 | – | – | – | – | – | – | – | – | – | – | – | – |
| SXT | N | – | – | – | – | – | 5 | – | – | 1 | – | 21 | – | 78 | – | 59 | – | 19 | 4 | 5 | 11 | 22 | – | 31 | 19 | – | – | – | – | – | – |
|  | Cum. % | – | – | – | – | – | 1.8 | – | – | 2.2 | – | 9.8 | – | 38.2 | – | 59.6 | – | 66.5 | 68.0 | 69.8 | 73.8 | 81.8 | – | 93.1 | 100 | – | – | – | – | – | – |
|  | % | – | – | – | – | – | 1.8 | – | – | 0.4 | – | 7.6 | – | 28.4 | – | 21.5 | – | 6.9 | 1.5 | 1.8 | 4.0 | 8.0 | – | 11.3 | 6.9 | – | – | – | – | – | – |

–, not applicable; AMC, amoxicillin/clavulanic acid; AMX, amoxicillin; Antim., antimicrobial; AZM, azithromycin; CDN, cefdinir; CEC, cefaclor; CFM, cefixime; CLR, clarithromycin; CPD, cefpodoxime; CRO, ceftriaxone; CTB, ceftibuten; CTX, cefotaxime; Cum., cumulative; CXM, cefuroxime; DOX, doxycycline; ERY, erythromycin; LVX, levofloxacin; MXF, moxifloxacin; PEN, penicillin; SXT, trimethoprim/sulfamethoxazole; TET, tetracycline.

Bold vertical bars in table correspond to the CLSI-susceptible breakpoints.
